# Theoretical Insights into the Impact of Pyrrole and Imidazole Substituents on the BODIPY Chromophore

**DOI:** 10.3390/molecules30102209

**Published:** 2025-05-18

**Authors:** Patrycja Piękoś, Paweł Lipkowski, Wim Dehaen, Robert Wieczorek, Aleksander Filarowski

**Affiliations:** 1 Faculty of Chemistry, University of Wroclaw, F. Joliot-Curie 14, 50-383 Wrocław, Poland; robert.wieczorek@uwr.edu.pl; 2 Department of Physical and Quantum Chemistry, Wrocław University of Science and Technology, Wybrzeże Wyspiańskiego 27, 50-370 Wrocław, Poland; pawel.lipkowski@pwr.edu.pl; 3 Department of Chemistry, KU Leuven, Celestijnenlaan 200f-bus 02404, 3001 Leuven, Belgium; wim.dehaen@kuleuven.be

**Keywords:** BODIPY dye, protonation, deprotonation, DFT, TD-DFT

## Abstract

This paper concerns the in silico studies of the influence of heterocyclic substituents as well as their protonated and deprotonated forms on the spectral characteristics of BODIPY (4,4-difluoro-4-bora-3a,4a-diaza-*s*-indacene) dyes. Computational studies were carried out in order to reveal the most effective method of modeling of the spectral features of fluorescent BODIPY dyes. To perform these studies, the pyrrole and imidazole derivatives of BODIPY dyes were selected, and their spectral features were investigated with DFT and TD-DFT calculations. The calculations showed that the deprotonation of the substituents leads to a bathochromic shift of the calculated absorption wavelength, while the protonation (imidazole derivative) brings about a hypsochromic shift with respect to the neutral form of the dye. The calculated spectral characteristics, considering the influence of the solvent polarity (PCM model), were correlated with the $E_{T}^{N}$ solvatochromic parameter. These correlations show that the increase in the solvent polarity causes a hypsochromic shift of the calculated absorption and emission wavelengths, whereas the bathochromic shift of the wavelengths is observed for the protonated form.

## 1. Introduction

This paper focuses on the theoretical studies of fluorescent BODIPY dyes [1,2,3,4,5,6,7]. These dyes have gained extreme popularity in the recent decade. The very first serendipitous synthesis of BODIPY dye by Treibs and Kreuzer [8] was given little attention by the community. However, the development of photo-technologies, enabling investigation of excited states, has aroused a genuine interest in fluorescent dyes. The popularity of fluorescent BODIPY dyes has been reinforced by their versatile prospective applications in medicine [9,10,11,12,13,14,15], biological imaging [16,17,18,19,20,21], antibacterial photodynamic therapy [22,23,24,25,26,27], chemistry, and technology [28,29,30,31]. One of the major advantages is the modification of the spectral properties by means of organic design, including pre- and post functionalization [32,33,34,35]. In later works, extensive and thorough studies have revealed their ability to serve as chemosensors of the environment polarity [36,37,38], pH [39,40,41,42,43], ions [44], the development of living organisms [16,18], and cancer tumors [11,15]. A key research direction lays in the design of novel materials in optoelectronics, based on the application of BODIPY dyes [44,45].

**Figure 1:**
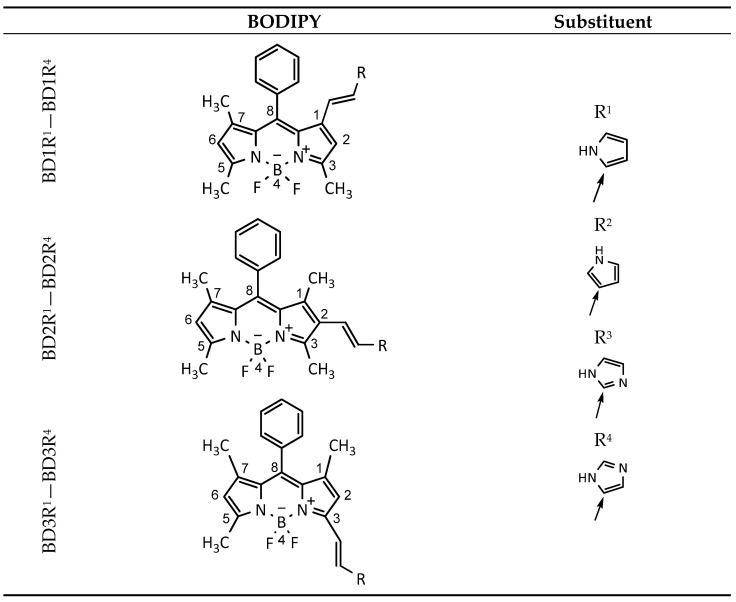
Structures of the studied molecules.

In light of the abovementioned facts, the theoretical elaboration of this type of dyes is definitely important. Numerous papers [46,47,48,49,50,51,52,53,54,55,56] deal with the successful use of the TD-DFT method in the description and analysis of spectral characteristics of a variety of fluorescent BODIPY dyes. Despite the deep theoretical development of these dyes, there is still an urgent need for theoretical studies of the role of the protonation and deprotonation of pyrrole and imidazole substituents in different positions of BODIPY dye (Figure 1). The rationale of these compounds relies on the BODIPY chromophore, which possesses unique spectral properties, and the imidazole and pyrrole substituents that actively participate in biological processes [57,58]. Of importance is π-electronic coupling between the heterocyclic substituent and the chromophore via the vinyl bridge, which transmits a signal from either the protonated or deprotonated substituent to the BODIPY core. These theoretical studies dwell on the fluorescent BODIPY dyes with pyrrole and imidazole substituents, which can be obtained by organic synthesis. Similar fluorescent BODIPY dyes with these substituents were presented only once before [59].

In this work, the research methodology was followed as explained below. Initially, a full optimization of the structures of the studied molecules was performed for neutral, protonated, and deprotonated substituents. Next, the calculations of the electronic transitions were performed, and the absorption and emission wavelengths were calculated, proceeded by the calculation of the highest occupied molecular orbital (HOMO) and the lowest unoccupied molecular orbital (LUMO). The calculations gave a way to the conformational analysis and the estimation of the influence of both the protonation and deprotonation of the substituent and the solvent polarity on the spectral parameters of the dyes. Such methodology is expected to enable the design of novel fluorescent dyes with desired spectral features.

## 2. Results and Discussion

**Figure 2:**
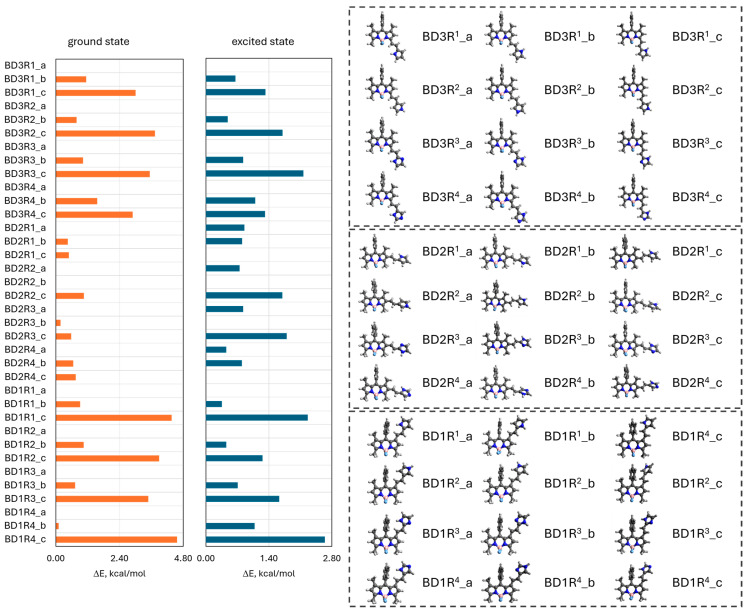
The optimized structures of conformers and relative energy (ΔE = E_min_ − E_i_) of studied dyes calculated with M062x/6-31+G(d,p) and TD-M062x/6-31+G(d,p) methods for full optimization parameters of the molecule at ground and excited states, respectively.

Conformational analysis for the ground and excited states was carried out for all the studied BODIPY derivatives and their protonated and deprotonated states. The completed DFT and TD-DFT calculations found the structures of the molecules with 1- and 3- substituents to be mostly flat (the planes between the BODIPY core and the substituents are considered here). This result indicates a significant π-electronic coupling between either the pyrrole or imidazole substituent and the BODIPY core, which holds these fragments in one plane. However, the molecules with substituents in the 2 position are not flat due to turning of either the pyrrole or imidazole fragment. Such a phenomenon is caused by the steric hindrance of the methyl groups in the 1 and 3 positions on the vinyl bridge. Based on the above observations, the structure of the studied molecules is influenced by π-electronic coupling and steric hindrance. The calculations, performed for all possible conformers, showed but a small difference in the energies between them in the ground and excited states (ΔE_i_ less than 4 kcal/mol; Figure 2 and Table S1 (Supplementary Materials, SM)). It is important to mention a significant role of the NH–π and hydrogen bond in the stabilization of the 1-derivatives’ structure. In terms of the conformational analysis for the excited state, TD-DFT calculations showed a similar trend. Notably, the benzene ring in the 8 position was not analyzed since in all the molecules, it is placed almost perpendicularly to the difluoroboradiazaindacene plane due to strong steric hindrance of two neighboring methyl groups in the 1 and 7 positions.

This section deals with the analysis of the shifts of the calculated absorption and emission wavelengths depending on both the type of the substituent (pyrrole or imidazole) and the substituent position (1, 2, or 3) (Table 1 and Table S1). This analysis was accomplished based on the data calculated by the TD-DFT method using the **SS** (state-specific) and **LR** (linear-response) approaches for chloroform. When it comes to the influence of the studied R^1^–R^4^ substituents in either 1, 2, or 3 positions on the calculated absorption and emission wavelengths, the following tendency is observed: the substitution of the 2-pyrrolyl substituent (R^1^) for either 3-pyrrolyl (R^2^), 2-imidazylyl (R^3^), or 4-imidazolyl (R^4^) mostly leads to a hypochromic shift of these wavelengths (Table 1). As for the influence of the R^1^–R^4^ substituents in different positions, the alteration of the substitution position from 3 to 1 (3→2; 3→1) for the R^1^ derivative brings about a bathochromic shift of the calculated absorption and emission wavelengths (Table 1).

Notably, the difference between the positions of the absorption or emission wavelengths for the isomeric pyrrole derivatives (R^1^ and R^2^) is distinctive, whereas for the imidazole derivatives, it is not significant (R^3^ and R^4^) (Table 1). The presented computational results show that the influence of the substituent (electronic and steric effects) on the spectral characteristics of the studied dyes is rather complicated. The substituents in the 2 position (under strong steric hindrance of the neighboring methyl groups) make the vinyl group go out of the plane despite π-electronic conjugation between the chromophore core and the substituent. Nevertheless, this picture is different for the excited state, when the π-electronic conjugation between the fragments is increasing, resulting in flattening of the molecule. Notably, these substitutions also evoke a bathochromic shift of the absorption and emission wavelengths in comparison to the bands of 1,3,5,7-tetramethyl-8-phenyl-4,4-difluoroboradiazaindacene (Table 1).

**Figure 3:**
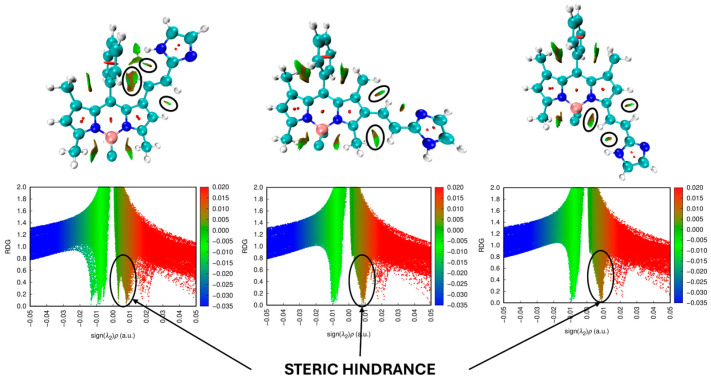
Non-covalent interactions (NCI) plots and RDG scatter plots of studied BODIPY dyes. The region of the non-covalent repulsion between the substituent and the BODIPY core fragment are marked as black circles.

It is important that the influence of the protonation and deprotonation of the studied substituents on the spectral characteristics of the dyes appears to be the most unambiguous and efficient. The deprotonation of the neutral form of the studied dyes brings about a bathochromic shift of the absorption and emission wavelengths (Figure 3 and Table 1, Tables S2 and S3). This opposite acidochromic effect for BODIPY dyes was studied by Akkaya et al. [60,61,62] in dipyridine–BODIPY dye and elaborated in the theoretical work by Jacquemin et al. [46]. However, the protonation of the neutral form of the imidazole derivatives provokes a hypsochromic shift of the absorption and emission wavelengths. It should be noted that the protonation/deprotonation phenomenon finds its reflection in the Stokes’ shift ($\Delta \bar{\nu }$; Tables S2 and S3), which has been elaborated upon in experimental studies [25,37,38].

The deprotonation of the pyrrole and imidazole substituents causes changes in the Stokes’ shift, especially for the derivatives in the 2 position. This result appears to be a consequence of various activities of competing π-electronic conjugation and steric repulsion between the methyl groups and the vinyl bridge. This visible Stokes’ shift comes as a result of structural changes after the transition from the ground state (GS) to the excited (ES) one. As described above, π-electronic conjugation makes the structure of 2-derivatives more planar despite two-sided steric hindrance of methyl groups next to the vinyl bridge. This phenomenon is weaker for the 1- and 2-derivatives because of the absence of two-sided steric hindrance.

In order to show and analyze the regions of steric hindrance in the studied molecules, the calculations of non-covalent interaction (NCI) plots and reduced density gradient (RDG) scatter plots were carried out. It is important to note that the obtained results (in terms of the steric hindrance) were consistent for all derivatives; thus, the only analysis for one imidazole derivative is presented below (Figure 3). Data for all molecules are presented in SM (Figure S1). The visualization of the results of NCI calculations shows that between the substituent (in all the positions of substitution (1, 2, and 3)) and the fragments of the BODIPY core, there is repulsive non-covalent interaction. According to RDG scatter plots, the repulsive interaction is not very strong but is distinctive. The presented results of the NCI calculations confirm the logical conclusion about the steric effect influence on the molecule structure.

**Figure 4:**
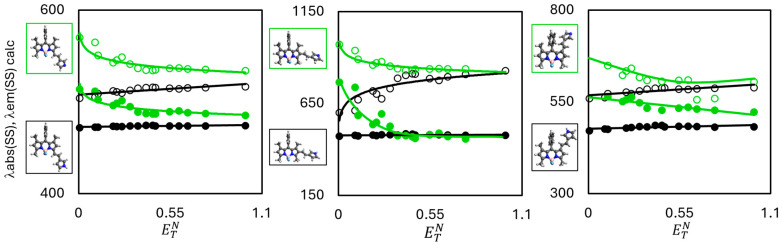
Dependencies of absorption (filled circles) and emission (open circles) wavelengths positions (obtained by the **SS** approach) on the $E_{T}^{N}$ solvatochromic parameter for neutral (black circles) and deprotonated (green circles) forms of studied dyes.

To estimate the effect of the solvent polarity on the calculated absorption and emission wavelengths, DFT and TD-DFT calculations in the **SS** and **LR** approaches were completed for a number of solvents (Table S6). The calculated spectral data were correlated with the Reichardt ($E_{T}^{N}$) [63] and SPP [64] solvatochromic parameters, which define the solvent polarity (Figure 4 and Figures S2–S7). Below is a summary of the results obtained by the calculations using the **SS** approach. The **SS** approach was chosen for its reliability from a theoretical viewpoint [46]. It should be also mentioned that the studies focus on the trends shown in the accomplished calculations but not on a perfect agreement of the calculated wavelengths with the experimental ones (in view of the shortage of experimental data).

**Figure 5:**
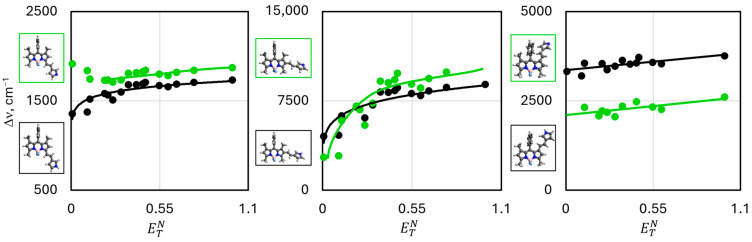
Dependency of the calculated Stokes’ shift ($∆\bar{\nu }$) on $E_{T}^{N}$ solvatochromic parameter for neutral (black circles) and protonated (green circles) forms of studied dyes.

The completed calculations revealed but a weak influence of the changing solvent polarity on the calculated wavelengths of the neutral form of the pyrrole derivatives (Figure 4, Figures S2 and S3). The calculations proved the increasing solvent polarity to cause a hypsochromic shift of the absorption and emission wavelengths of the neutral form. However, an opposite trend was observed for the protonated form of the dyes—the increase in the solvent polarity triggers an insignificant bathochromic shift of the absorption and emission wavelengths (Figure 4 and Figure S3); moreover, in the case of the 2-derivative, the emission bands undergo a significant bathochromic shift. Also, it is noteworthy that the polarity increase is accompanied by an increase in the Stokes’ shift (increasing $E_{T}^{N}$ and SPP solvatochromic parameters). Some irregularities, as observed for the 2-derivative, are caused by the competing effects, as explained above (Figure 5).

**Figure 6:**
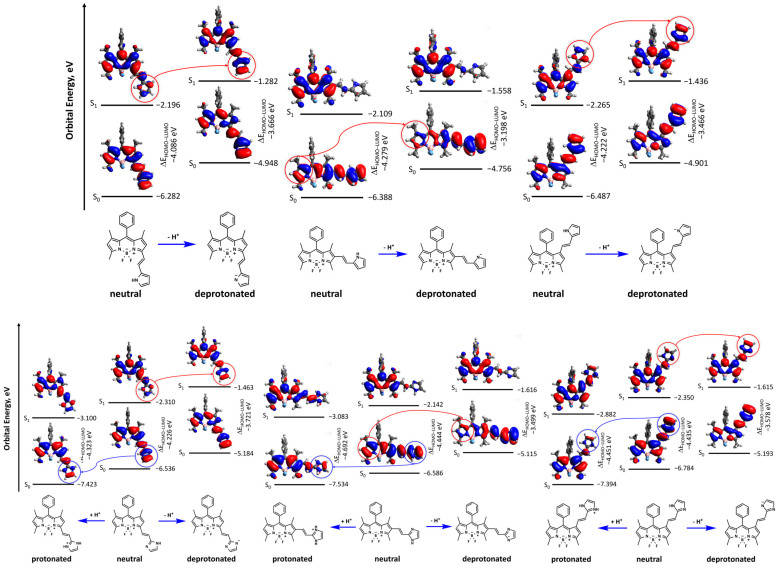
Energy levels and isosurfaces of selected isomers of dyes computed with M062x/6-31+G(d,p) method.

The theoretical and experimental studies of similar BODIPY dyes are presented in many papers [65,66,67]. According to the studies of the BODIPY dyes [68], the HOMO neutral form being somewhat higher compared to the LUMO of the protonated form and the LUMO of the neutral form being lower than the HOMO of the deprotonated form support a significant photoinduced charge transfer. This transfer emerges after the transition from the neutral form to the protonated or deprotonated one, which results in a visible fluorescence decrease. The studied dyes do not feature this phenomenon—the energies of the HOMO and LUMO of the neutral forms are extensively overlapping with the energies of the HOMO and LUMO of the protonated and deprotonated forms (Figure 6 and Figure S8). Supposedly, this result suggests that in the studied dyes, quenching of the fluorescence will be weak compared to the strong one observed in 8-hydroxyquinoline–BODIPY derivatives [68].

Notably, the HOMO and LUMO undergo changes on the BODIPY core and vinyl-pyrrole/imidazole fragments after the transition from the neutral form to either the protonated form or the deprotonated one. The comparison of the neutral form with the deprotonated one does not feature a visible change of the isosurface for the pyrrole and imidazole derivatives in the ground state (Figure 6 and Figure S8). Moreover, the GS→ES transition is accompanied by a significant change of the isosurface on the pyrrole and imidazole fragments (red circles on Figure 6 and Figure S8). This phenomenon is reflected in the bathochromic shift of the calculated absorption and emission wavelengths (Table 1).

As for the protonation of the neutral form of the imidazole derivatives, it provokes changing of the isosurface on the imidazole fragment (blue circles on Figure 6 and Figure S8). These changes are reflected in the hypsochromic shift of the calculated absorption and emission wavelengths (Table 1).

The electron density difference (EDD) plots, calculated for the neutral and deprotonated forms (Figure S9), show that the GS→ES transition causes the increase in electron density on the pyrrole and imidazole substituents and its decrease on the BODIPY core. This result points out the charge transfer from the BODIPY core to the substituent under the GS→ES transition. However, the imidazolium derivatives are not characterized by electron transfer between the BODIPY core and substituent due to the unchanged electron density on the substituent (Figure S9). In imidazolium derivatives, redistribution generally occurs on the BODIPY core. The abovementioned differences in the behavior of the electron density between the neutral/deprotonated and protonated BODIPY dyes manifest themselves in the changes of the absorption and emission bands since these bands are hypsochromically shifted relative to the bands of neutral and deprotonated forms.

## 3. Materials and Methods

The calculations were accomplished with the Gaussian 16 ver. C01 [69] program using 6-31+G(d,p) basis set [70] and M06-2X functionals [71]. The calculations were performed for the ground (density functional theory, DFT [72,73]) and excited (time-dependent density functional theory, TD-DFT [74]) states. It is worth noting that the TD-M062X/6-31+G(d,p) method is a reliable for calculations of the BODIPY dyes [75]. The absorption and emission electronic transitions were calculated for the solvents of different polarity. The contribution of solvent effects was calculated using polarizable continuum model (PCM) [76,77] by linear-response (**LR**) [78,79] and state-specific (**SS**) [80] approaches. The **LR** approach is computationally efficient for excited-state geometry optimization. However, this approach does not take into account the cavity polarization changes during the transition from the ground to the excited state [46,81]. Therefore, the self-consistent **SS** approach was also used, which obtains more accurate transition energies for solvated species. The D3-DFT method was used to involve the dispersion forces [82]. It is worth mentioning that a strong specific interrelation between the dye substituent and any additional molecule (a strong acid or a strong base, e.g., HCl or NaOH) was not the subject of this work. The software was also used to perform the isosurface analysis of the HOMO and LUMO orbitals [83]. The non-covalent interactions (NCI) analysis was performed using the MultiWFN package—ver. 3.8 [84]. The results were visualized with the Avogadro, VMD, and GaussView programs [85,86,87].

## 4. Conclusions

This work revealed the conformers with minimum energy (by means of DFT (M06-2X/6-31+G(d,p)) and TD-DFT (TD-M06-2X/6-31+G(d,p))), which were used for the further calculations. According to the calculations performed, the difference between the steric and conjugated effects in the ground and excited states heavily influences the Stokes’ shift. The studies showed that the deprotonation of the pyrrole and imidazole fragments of the studied molecules evokes the bathochromic shift. Conversely, the protonation of the imidazole fragment of these molecules leads to the hypsochromic shift. The results of the calculations confirm that increasing the solvent polarity intensifies the Stokes’ shift, both for the protonated and deprotonated forms.

Based on the calculated data of HOMO and LUMO, one can conclude that the deprotonation of the pyrrole and imidazole derivatives strongly changes the isosurface of the substituent in the excited state. Concerning the protonation of the imidazole derivatives, this phenomenon provokes the decrease in the HOMO and LUMO energies as well as neutralization (a slight change of the isosurface under the transition from the ground state to the excited one) of the imidazole fragment of the dye.

The quantum–mechanical calculations, conducted by the DFT and TD-DFT methods for the ground and excited states, demonstrated that the protonation and deprotonation influence the position of the absorption and emission wavelengths more efficiently compared to the changing position of substitution (1, 2, and 3) with the pyrrole and imidazole substituents.

## Figures and Tables

**Table 1:** Calculated absorption (λ_abs_, nm) and emission (λ_abs_, nm) wavelengths by M062X methods under **SS** approach in chloroform of studied dyes.

	λ_abs_			λ_em_		
Substituent/Position	3	2	1	3	2	1
Neutral form						
R^1^	484.70	496.27	491.49	525.07	704.23	575.29
R^2^	473.07	479.68	474.73	509.44	668.12	559.15
R^3^	467.69	453.90	445.39	504.96	581.83	532.58
R^4^	468.83	455.55	450.28	503.32	583.81	531.44
Deprotonated form						
R^1^	514.98	631.07	575.73	563.17	922.76	639.65
R^2^	501.43	593.24	562.58	548.48	877.08	641.19
R^3^	499.89	547.79	541.60	548.5	820.45	618.86
R^4^	495.52	534.92	529.57	542.08	790.40	604.67
Protonated form						
R^3^	459.50	436.45	438.76	499.92	454.54	481.98
R^4^	456.87	443.04	432.91	486.92	464.74	466.32
1,3,5,7-Tetramethyl-8-phenyl-4,4-difluoroboradiazaindacene						
H	413.85			429.4		

## Data Availability

Data are contained within the article.

## Supplementary Materials

The following supporting information can be downloaded at https://www.mdpi.com/article/10.3390/molecules30102209/s1: Figure S1. Non-covalent interactions (NCI) plots and RDG scatter plots of studied BODIPY dyes; Figure S2: Dependency of absorption (filled squares) and emission (open squares) bands positions (obtained by **LR** approach) on $E_{T}^{N}$ solvatochromic parameter for neutral (black squares) and deprotonated (green squares) forms of studied dyes; Figure S3: Dependency of absorption (filled circles) and emission (open circles) bands positions (obtained by **SS** approach) on SPP solvatochromic parameter for neutral (filled circles) and deprotonated (green circles) forms of studied dyes; Figure S4: Dependency of calculated Stokes’ shift ($∆\bar{\nu }$) (**LR** approach) on $E_{T}^{N}$ solvatochromic parameter for neutral (green squares) and protonated (black squares) forms of studied dyes; Figure S5: Dependency of calculated Stokes’ shift ($∆\bar{\nu }$) (**SS** approach) on SPP solvatochromic parameter for neutral (yellow circles) and deprotonated (blue circles) forms of studied dyes; Figure S6: Dependency of absorption (filled squares) and emission (open squares) wavelengths (obtained by **LR** approach) on SPP solvatochromic parameter for neutral (blue squares) and deprotonated (green squares) forms of studied dyes; Figure S7: Dependency of calculated Stokes’ shift ($∆\bar{\nu }$) (**LR** approach) on SPP solvatochromic parameter for neutral (green squares) and deprotonated (blue squares) forms of studied dyes; Figure S8. Energy levels and isosurfaces of dyes computed with M062x/6-31+G(d,p) method; Figure S9: Electron density difference (EDD) plots between the excited state and ground state for studied dyes and its protonated and deprotonated forms. The red or blue zones indicate increase or decrease in density, respectively, upon electronic transition; Table S1: Calculated spectroscopic data (λ_abs_ and λ_em_, nm) of conformers obtained for global energy minimum of studied dyes in chloroform and relative energy at ground (ΔE_gr.st_, kcal/mol) and excited (ΔE_ex.st_, kcal/mol) states (M062x/6-31+G(d,p)), where upper lines refer to data obtained by **SS** approach, and bottom lines refer to data obtained by **LR** approach; Table S2: Calculated spectroscopic data (λ_abs_ and λ_em_, nm; $∆\bar{v}$, cm^−1^) of neutral and deprotonated forms of pyrrole derivatives in chloroform (M062x/6-31+G(d,p)), where upper lines refer to data obtained by **SS** approach, and bottom lines refer to data obtained by **LR** approach; Table S3: Calculated spectroscopic data (λ_abs_ and λ_em_, nm; $∆\bar{v}$, cm^−1^) of neutral, protonated, and deprotonated forms of imidazole derivatives in chloroform (M062x/6-31+G(d,p)), where upper lines refer to data obtained by **SS** approach, and bottom lines refer to data obtained by **LR** approach; Table S4: Frontier molecular orbitals of studied dyes and orbital energy (eV) for ground and excited states; Table S5: Calculated spectroscopic data (λ_abs_ and λ_em_, nm; $∆\bar{v}$, cm^−1^) of neutral and deprotonated forms of pyrrole derivatives in chloroform (M062x/6-31+G(d,p)), where upper lines refer to data obtained by **SS** approach, and bottom lines refer to data obtained by **LR** approach; Table S6: Calculated spectroscopic data of conformers referring to global energy minimum of studied dye for different solvents (M062x/6-31+G(d,p)).

## Abbreviations

The following abbreviations are used in this manuscript:

BODIPY	4,4-difluoro-4-bora-3a,4a-diaza-*s*-indacene
DFT	Density functional theory
TD-DFT	Time-dependent density functional theory
NCI	Non-covalent interaction
RDG	Reduced density gradient
GS	Ground state
ES	Excited state
SM	Supplementary Materials
HOMO	Highest occupied molecular orbital
LUMO	Lowest unoccupied molecular orbital
**LR**	Linear-response
**SS**	State-specific
PCM	Polarizable continuum model
BD1R1	1-(2-(1H-pyrrol-2-yl)vinyl)-3,5,7-trimethylphenyl BODIPY
BD1R2	1-(2-(1H-pyrrol-3-yl)vinyl)-3,5,7-trimethylphenyl BODIPY
BD1R3	1-(2-(1H-imidazol-2-yl)vinyl)-3,5,7-trimethylphenyl BODIPY
BD1R4	1-(2-(1H-imidazol-5-yl)vinyl)-3,5,7-trimethylphenyl BODIPY
BD2R1	2-(2-(1H-pyrrol-2-yl)vinyl)-1,3,5,7-tetramethylphenyl BODIPY
BD2R2	2-(2-(1H-pyrrol-3-yl)vinyl)-1,3,5,7-tetramethylphenyl BODIPY
BD2R3	2-(2-(1H-imidazol-2-yl)vinyl)-1,3,5,7-tetramethylphenyl BODIPY
BD2R4	2-(2-(1H-imidazol-5-yl)vinyl)-1,3,5,7-tetramethylphenyl BODIPY
BD3R1	3-(2-(1H-pyrrol-2-yl)vinyl)-1,5,7-trimethylphenyl BODIPY
BD3R2	3-(2-(1H-pyrrol-3-yl)vinyl)-1,5,7-trimethylphenyl BODIPY
BD3R3	3-(2-(1H-imidazol-2-yl)vinyl)-1,5,7-trimethylphenyl BODIPY
BD3R4	3-(2-(1H-imidazol-5-yl)vinyl)-1,5,7-trimethylphenyl BODIPY
