# Supplementary material for: Theoretical Insights into the Impact of Pyrrole and Imidazole Substituents on the BODIPY Chromophore
Source: Molecules. 2025 May 18;30(10):2209. doi: 10.3390/molecules30102209 (PMC12114514; doi:10.3390/molecules30102209)
Supplement: Supplementary file 1 [file molecules-30-02209-s001.zip › molecules-3626043-supplementary.pdf]

Supplementary material.

Theoretical insights into the impact of pyrrole and imidazole substituents on the BODIPY chromophore

Patrycja Piękoś [1,*], Paweł Lipkowski [2], Wim Dehaen [3], Robert Wieczorek [1] and Aleksander Filarowski [1,*]

[1] *Faculty of Chemistry, University of Wroclaw, F. Joliot-Curie 14, 50-383 Wrocław, Poland*
[2] *Department of Physical and Quantum Chemistry, Wrocław University of Science and Technology, Wybrzeże Wyspiańskiego 27, 50-370 Wrocław, Poland*

[3] *Department of Chemistry, KU Leuven, Celestijnenlaan 200f-bus 02404, 3001 Leuven, Belgium*

Figure S1. Non-covalent interactions (NCI) plots and RDG scatter plots of studied BODIPY dyes.

<table>
<tr><th>neutral form</th><th>deprotonated form</th><th>protonated form</th></tr>
<tr><td colspan="3">1-position of substitution</td></tr>
<tr><td colspan="3">Substituent R¹</td></tr>
<tr><td>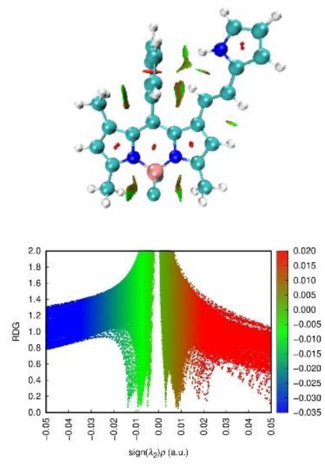
</td><td>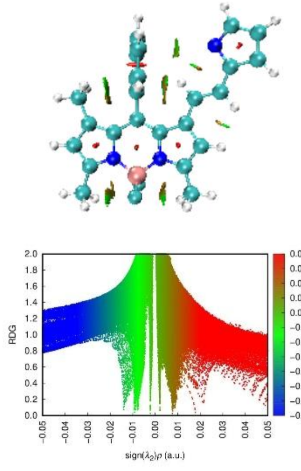
</td><td></td></tr>
<tr><td colspan="3">Substituent R²</td></tr>
<tr><td>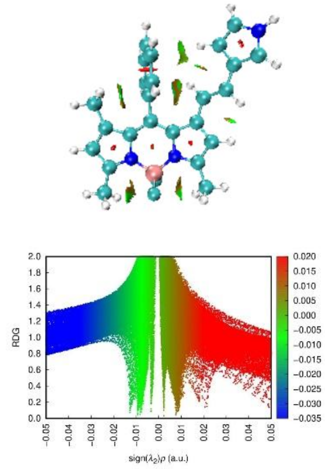
</td><td>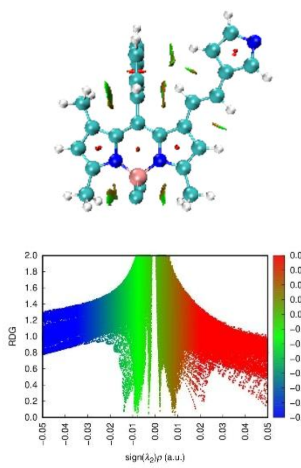
</td><td></td></tr>
</table>

**Substituent R^3**		
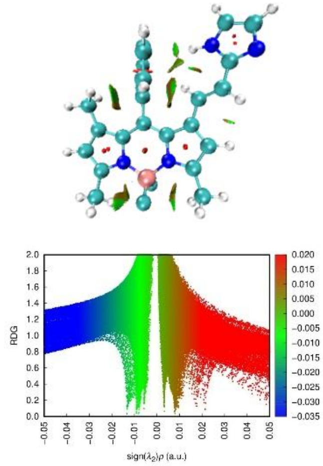	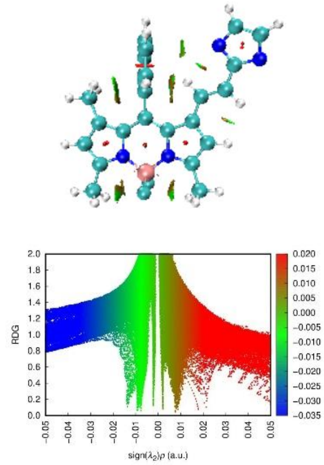	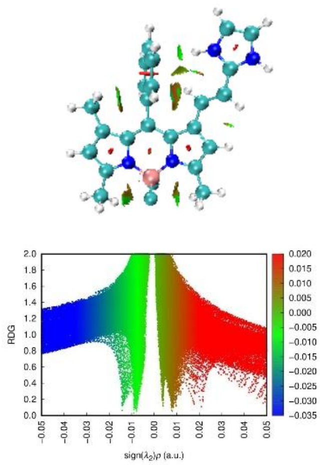
**Substituent R^4**		
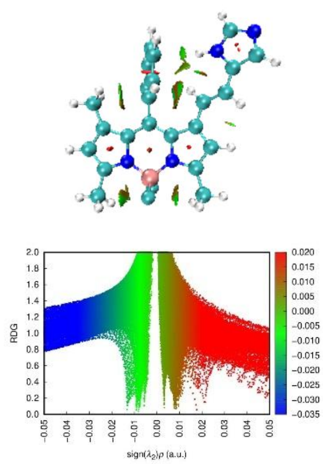	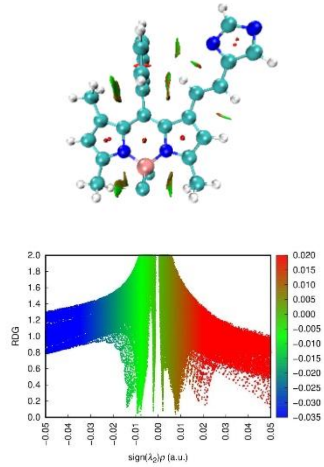	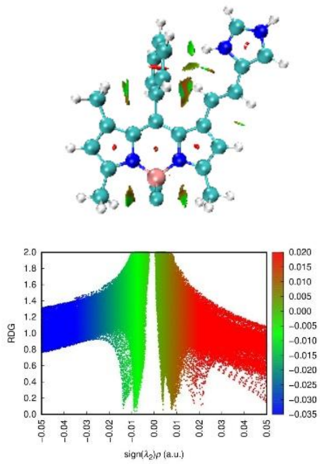

2-position of substitution

**Substituent R^1**		
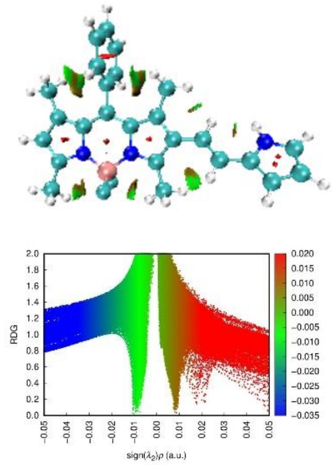	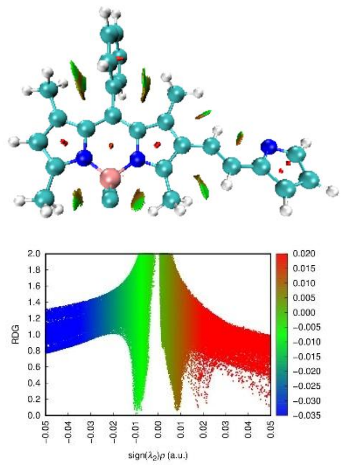	
**Substituent R^2**		

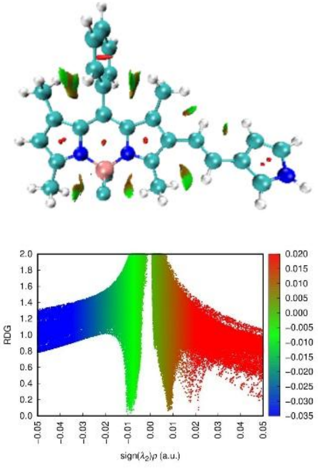	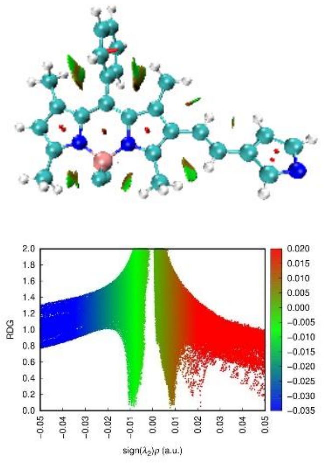	
**Substituent R³**		
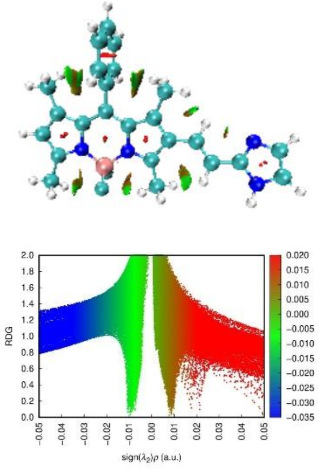	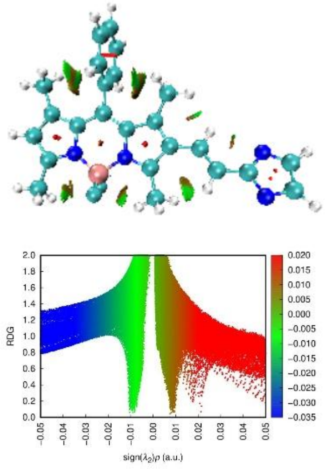	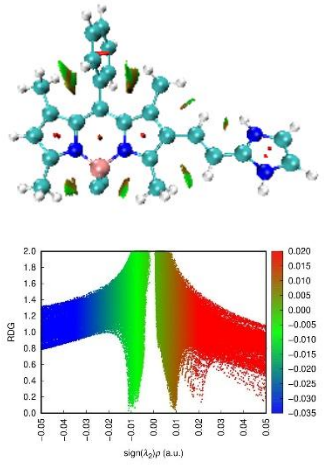
**Substituent R⁴**		
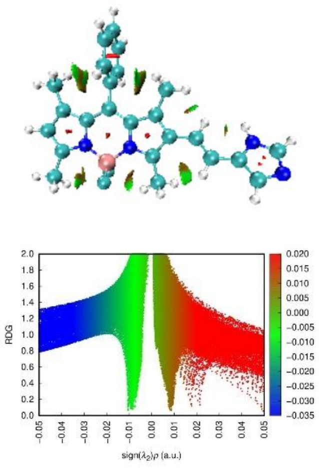	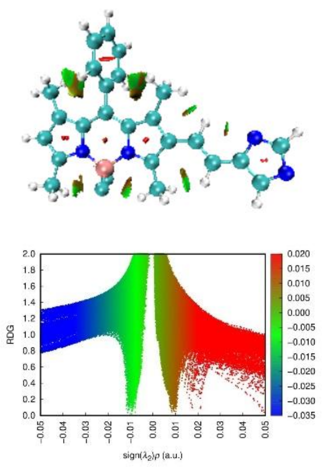	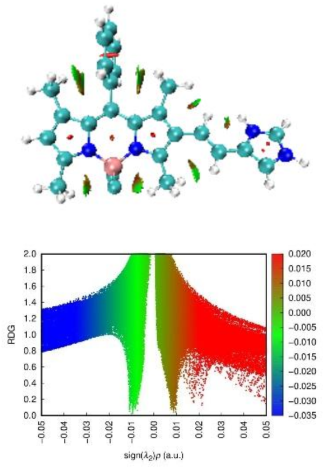

3-position of substitution

Substituent R¹

<table>
<tr><td>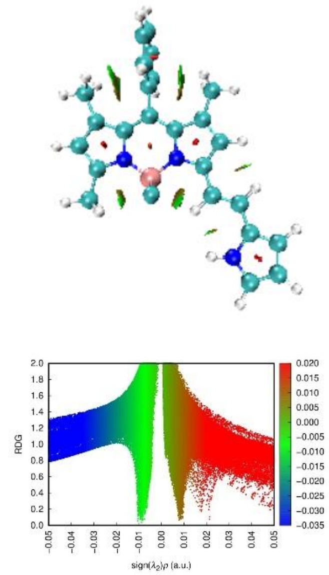
</td><td>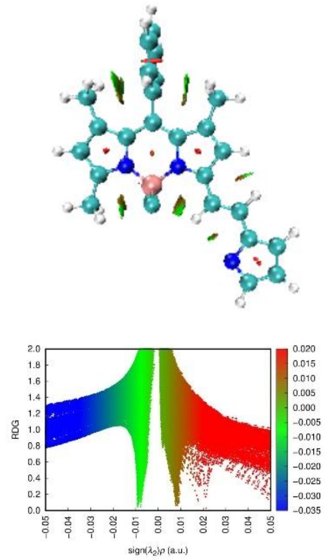
</td><td></td></tr>
<tr><td colspan="3">Substituent R²</td></tr>
<tr><td>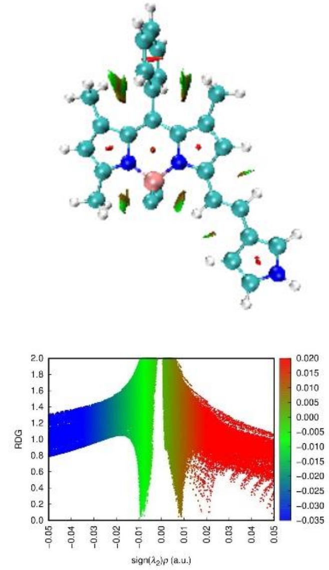
</td><td>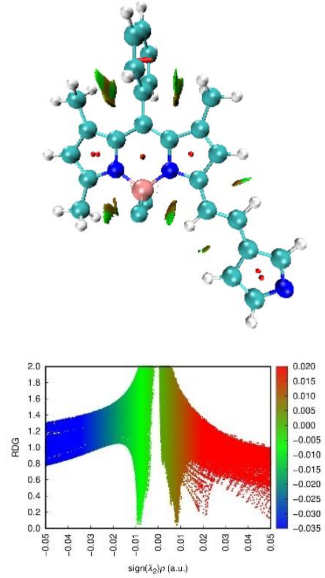
</td><td></td></tr>
<tr><td colspan="3">Substituent R³</td></tr>
<tr><td>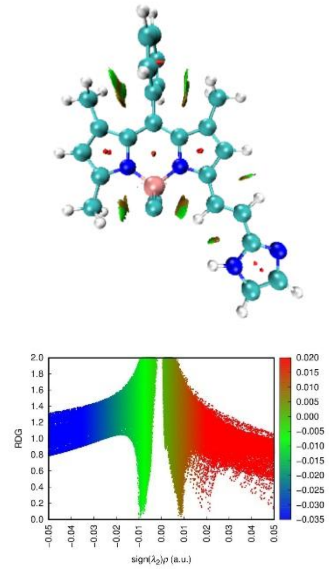
</td><td>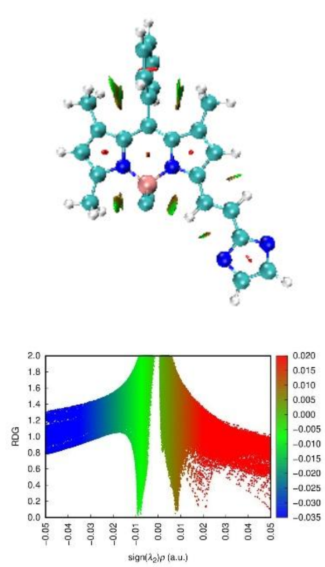
</td><td>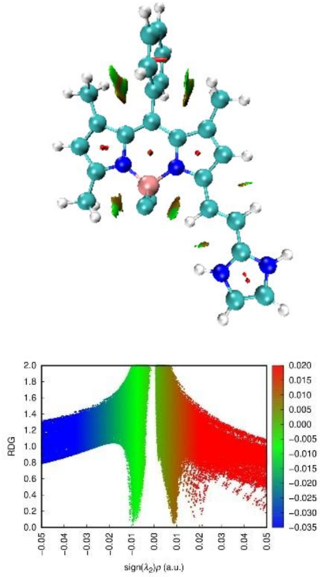
</td></tr>
</table>

Substituent R^4		
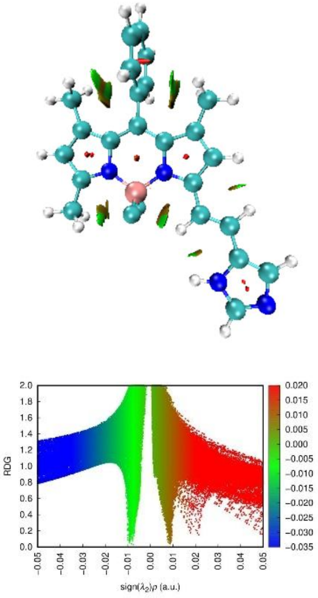	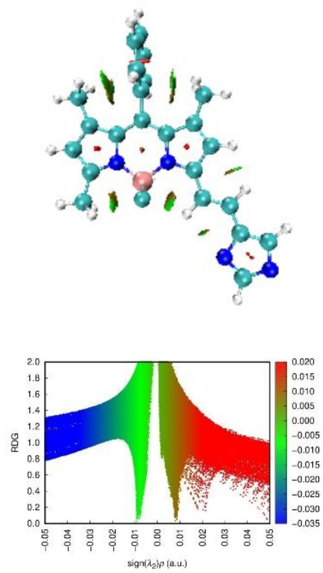	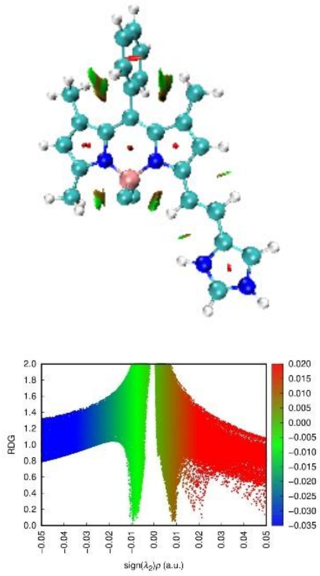

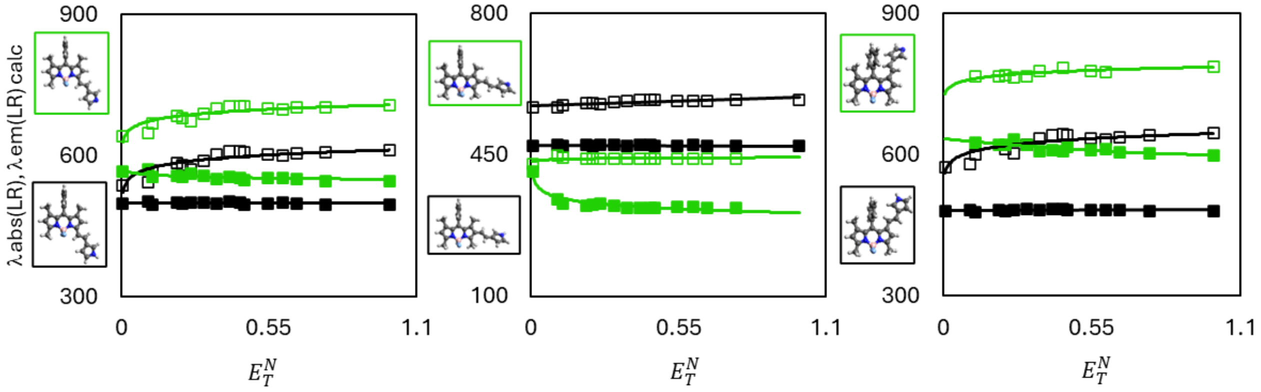

Figure S2. Dependency of absorption (filled squares) and emission (open squares) wavelengths (obtained by **LR** approach) on E_T^N solvatochromic parameter for neutral (black squares) and deprotonated (green squares) forms of studied dyes.

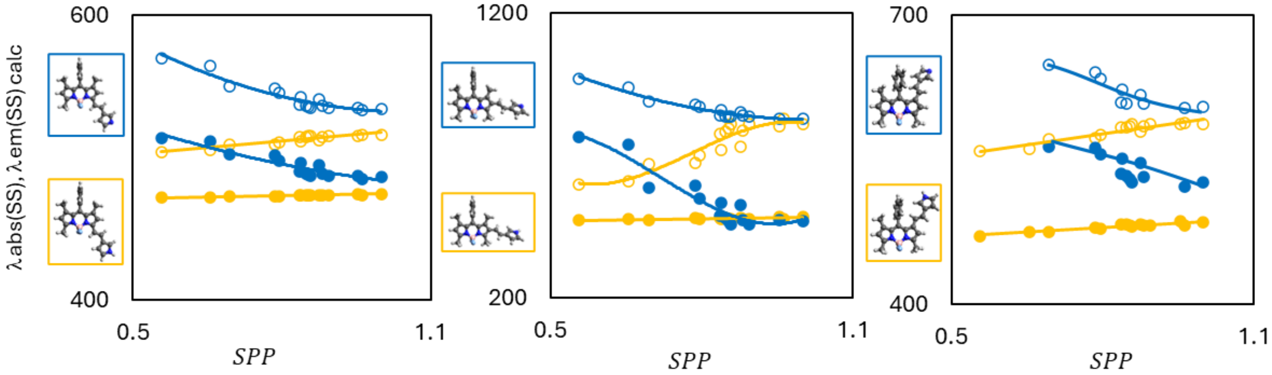

Figure S3. Dependency of absorption (filled circles) and emission (open circles) wavelengths (obtained by **SS** approach) on SPP solvatochromic parameter for neutral (filled circles) and deprotonated (green circles) forms of studied dyes.

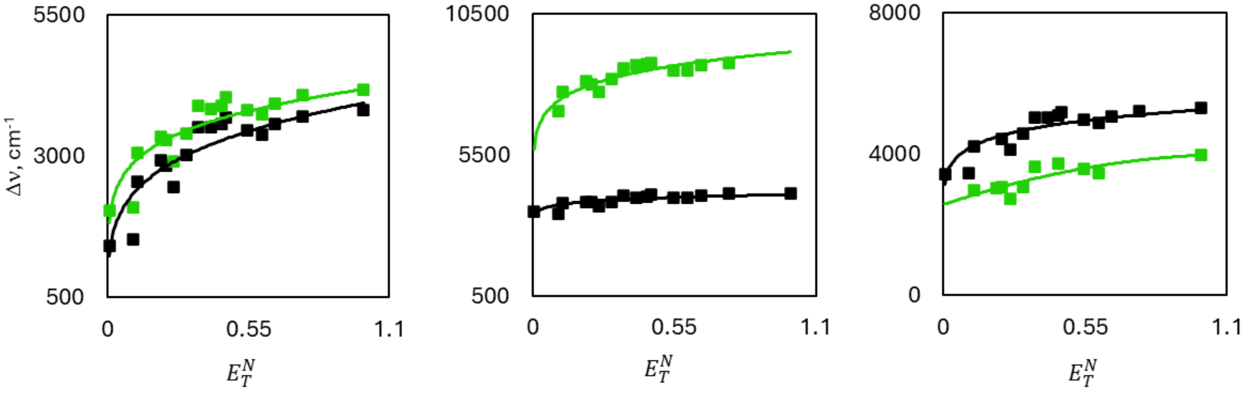

Figure S4. Dependency of calculated Stokes' shift ($\Delta\bar{\nu}$) (**LR** approach) on E_T^N solvatochromic parameter for neutral (green squares) and protonated (black squares) forms of studied dyes.

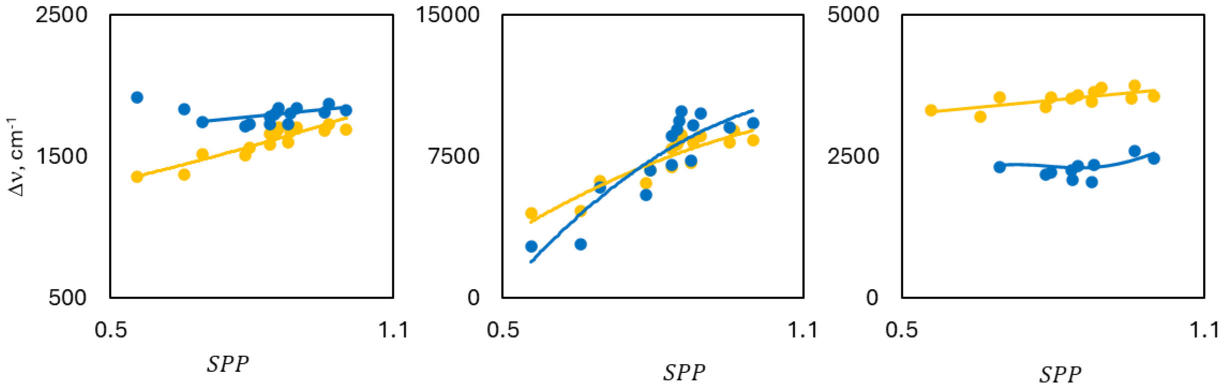

Figure S5. Dependency of calculated Stokes' shift ($\Delta\bar{\nu}$) (**SS** approach) on SPP solvatochromic parameter for neutral (yellow circles) and deprotonated (blue circles) forms of studied dyes.

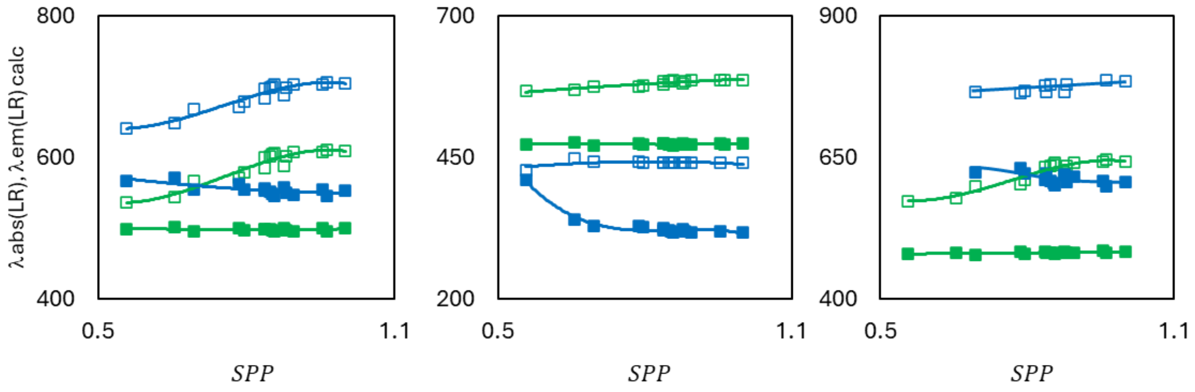

Figure S6. Dependency of absorption (filled squares) and emission (open squares) wavelengths (obtained by **LR** approach) on SPP solvatochromic parameter for neutral (blue squares) and deprotonated (green squares) forms of studied dyes.

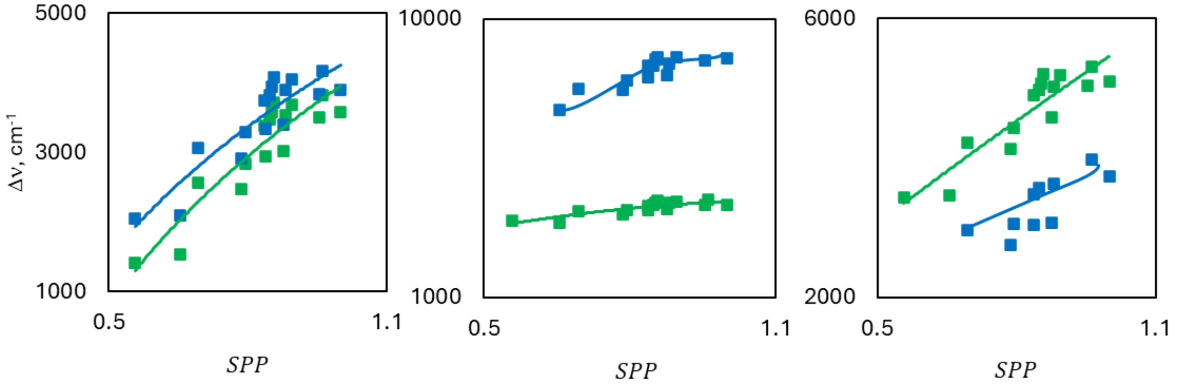

Figure S7. Dependency of calculated Stokes' shift ($\Delta\bar{\nu}$) (**LR** approach) on SPP solvatochromic parameter for neutral (green squares) and deprotonated (blue squares) forms of studied dyes.

Figure S8. Energy levels and isosurfaces of dyes computed with M062x/6-31+G(d,p) method.

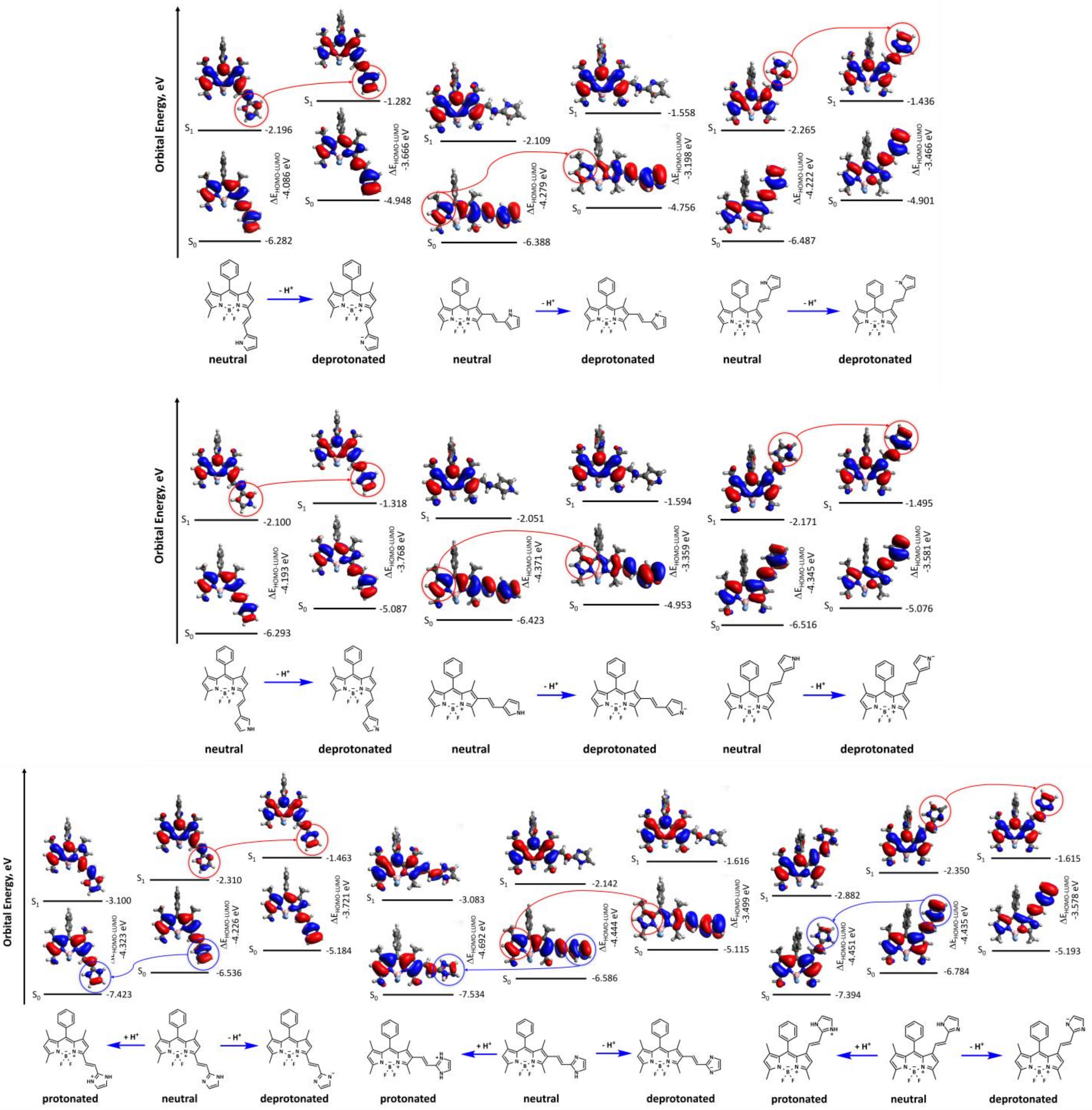

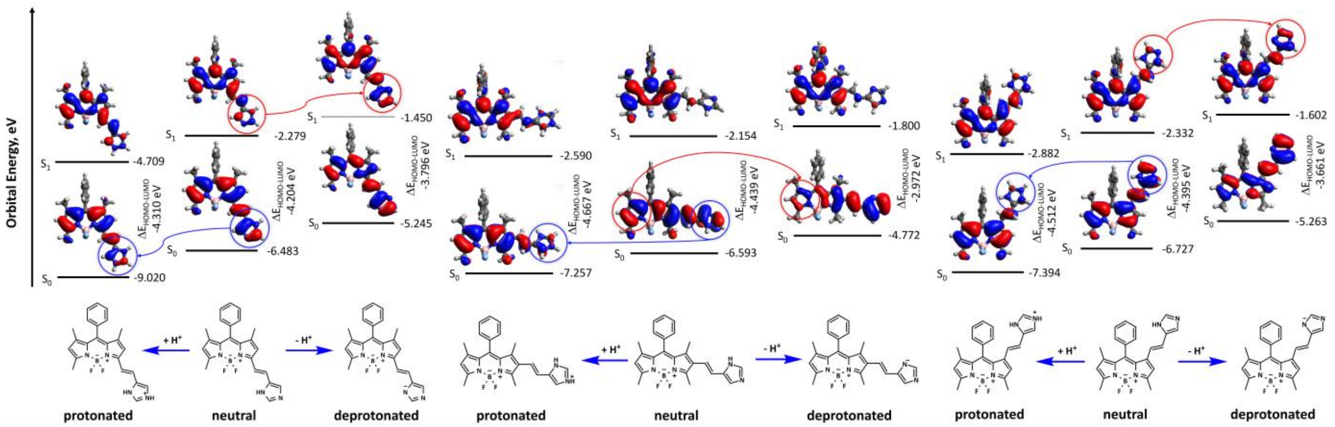
Orbital Energy, eV
S1 -4.709
S0 -9.020
ΔE HOMO-LUMO -4.310 eV
S1 -2.279
S0 -6.483
ΔE HOMO-LUMO -4.204 eV
S1 -1.450
S0 -5.245
ΔE HOMO-LUMO -3.796 eV
S1 -2.590
S0 -7.257
ΔE HOMO-LUMO -4.667 eV
S1 -2.154
S0 -6.593
ΔE HOMO-LUMO -4.439 eV
S1 -1.800
S0 -4.772
ΔE HOMO-LUMO -2.972 eV
S1 -2.882
S0 -7.394
ΔE HOMO-LUMO -4.512 eV
S1 -2.332
S0 -6.727
ΔE HOMO-LUMO -4.395 eV
S1 -1.602
S0 -5.263
ΔE HOMO-LUMO -3.661 eV
+ H+
- H+
protonated
neutral
deprotonated
protonated
neutral
deprotonated
protonated
neutral
deprotonated

Figure S9. Electron density difference (EDD) plots between the excited state and ground state for studied dyes and its protonated and deprotonated forms. The red or blue zones indicate increase or decrease of density, respectively, upon electronic transition.

neutral form	deprotonated form	protonated form
**1-position of substitution**		
**Substituent R¹**		
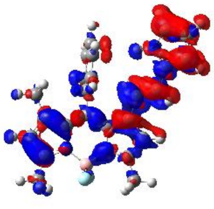	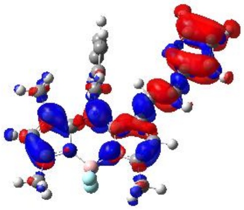	
**Substituent R²**		
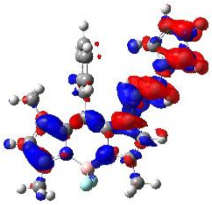	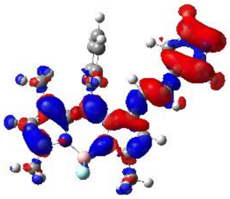	
**Substituent R³**		
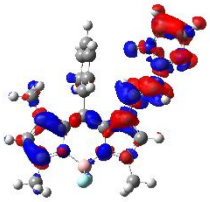	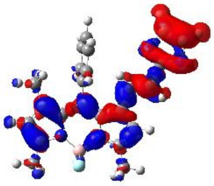	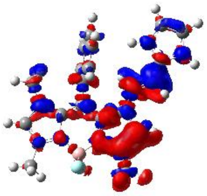
**Substituent R⁴**		
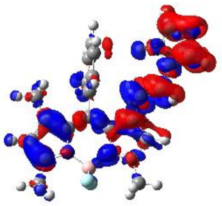	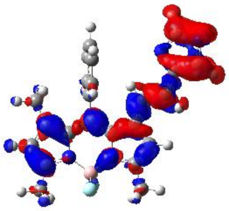	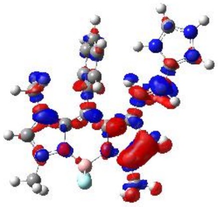
**2-position of substitution**		
**Substituent R¹**		
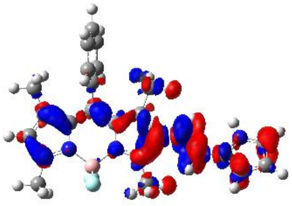	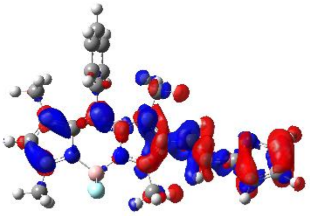	
**Substituent R²**		
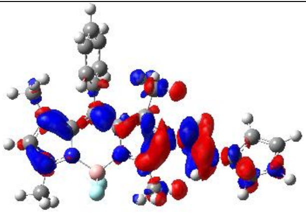	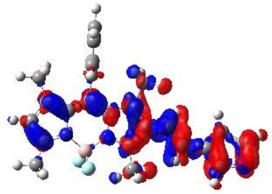	

**Substituent R³**		
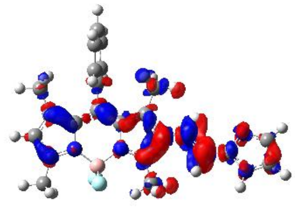	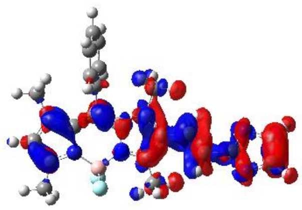	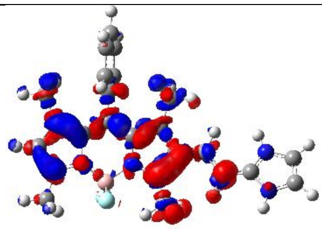
**Substituent R⁴**		
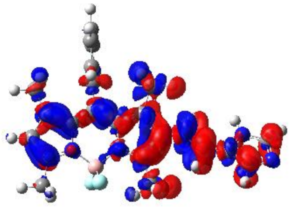	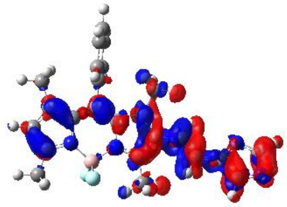	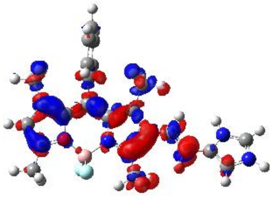
**3-position of substitution**		
**Substituent R¹**		
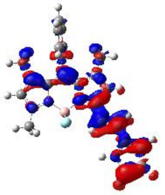	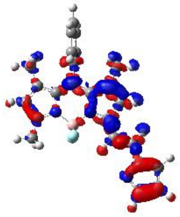	
**Substituent R²**		
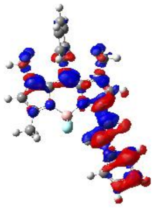	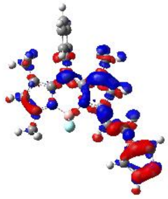	
**Substituent R³**		
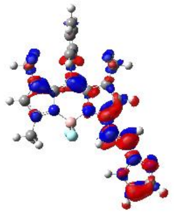	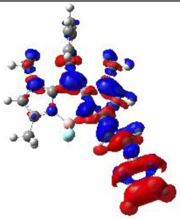	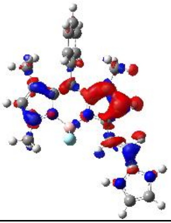
**Substituent R⁴**		
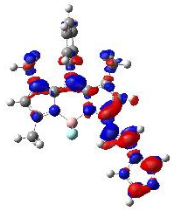	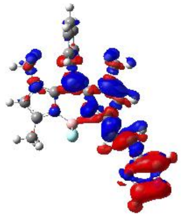	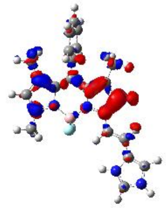

Table S1. Calculated spectroscopic data (λ_{abs} and λ_{em}, nm) of conformers obtained for global energy minimum of studied dyes in chloroform and relative energy at ground ($\Delta E_{gr.st}$, kcal/mol) and excited ($\Delta E_{ex.st}$, kcal/mol) states (M062x/6-31+G(d,p)). Upper lines refer to data obtained by **SS** approach; bottom lines refer to data obtained by **LR** approach.

Conf.	ground state	λ_{abs}, nm	excited state	λ_{em}, nm	ΔE_{grte}	$\Delta E_{excited\ state}$
**Substitution R^1**						
BD1R^1_a	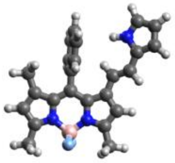	491.49<br>492.77	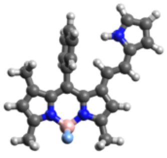	575.29<br>618.81	0.00	0.00
BD1R^1_b	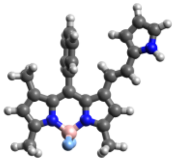	504.64<br>497.95	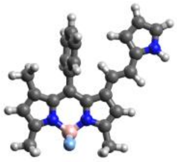	587.34<br>626.57	0.92	0.36
BD1R^1_c	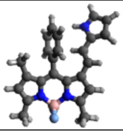	518.99<br>502.62	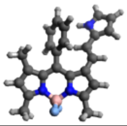	672.09<br>683.34	4.37	2.26
BD2R^1_a	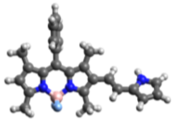	496.27<br>480.03	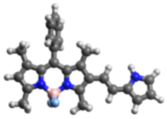	704.23<br>582.77	0.00	0.86
BD2R^1_b	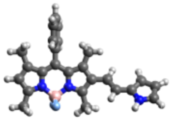	508.60<br>482.99	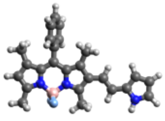	730.43<br>591.87	0.46	0.80
BD2R^1_c	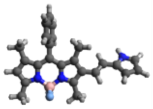	518.99<br>502.62	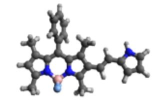	672.09<br>683.34	0.49	0.00
BD3R^1_a	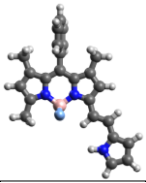	484.70<br>514.17	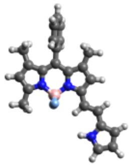	525.07<br>594.17	0.00	0.00
BD3R^1_b	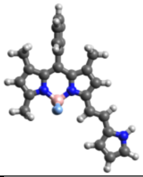	488.62<br>517.83	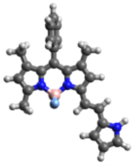	530.98<br>601.39	1.14	0.66
BD3R^1_c	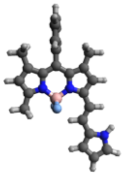	504.72<br>532.94	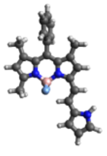	547.06<br>613.84	3.00	1.33
**Substitution R^2**						
BD1R^2_a	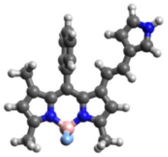	479.68<br>475.77	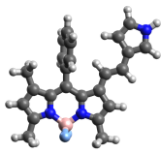	668.12<br>572.87	0.00	0.00

BD1R^2_b	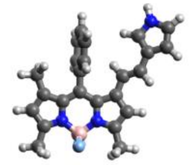	480.03<br>482.92	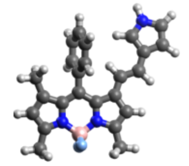	572.61<br>603.32	1.05	0.45
BD1R^2_c	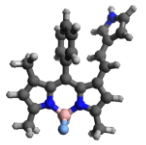	505.52<br>476.52	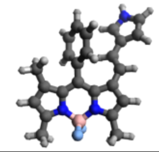	696.07<br>678.31	3.90	1.26
BD2R^2_a	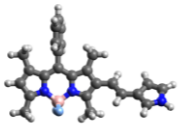	474.73<br>480.33	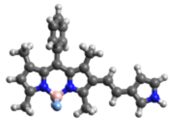	559.15<br>591.28	0.00	0.00
BD2R^2_b	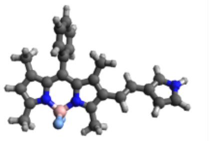	478.13<br>477.61	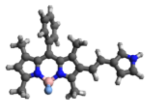	661.71<br>584.14	0.01	0.00
BD2R^2_c	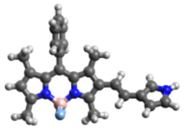	478.91<br>475.22	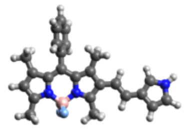	675.60<br>575.52	1.06	0.95
BD3R^2_a	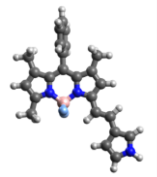	473.07<br>500.62	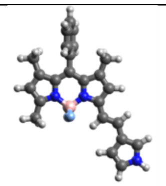	509.44<br>571.00	0.00	0.00
BD3R^2_b	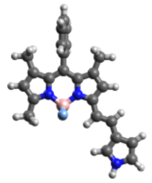	475.84<br>503.31	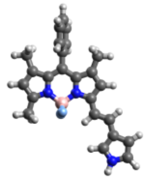	513.98<br>575.93	0.79	0.49
BD3R^2_c	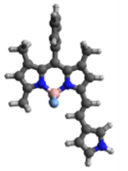	495.33<br>521.36	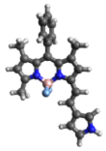	536.33<br>595.99	3.74	1.70
**Substitution R^3**						
BD1R^3_a	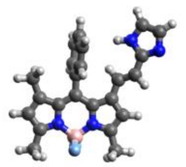	445.39<br>470.43	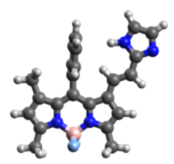	532.58<br>576.21	0.00	0.00
BD1R^3_b	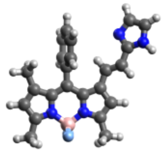	451.24<br>471.23	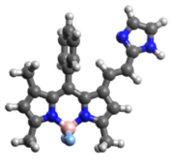	544.07<br>576.98	0.73	0.71

BD1R^3_c	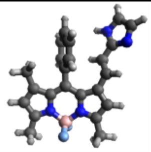	460.34<br>476.08	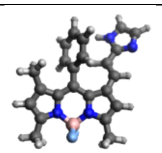	621.13<br>633.67	3.48	1.63
BD2R^3_a	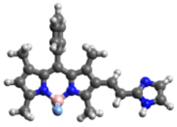	453.89<br>467.39	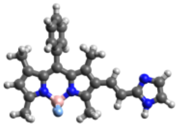	581.83<br>535.45	0.00	0.83
BD2R^3_b	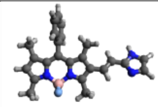	455.51<br>471.52	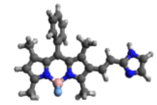	580.69<br>550.28	0.18	0.00
BD2R^3_c	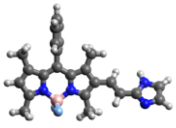	448.32<br>465.25	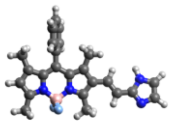	554.94<br>529.81	0.58	0.97
BD3R^3_a	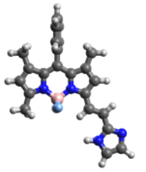	467.69<br>496.90	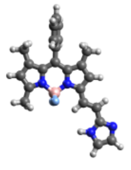	504.96<br>569.35	0.00	0.00
BD3R^3_b	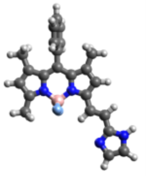	469.28<br>498.00	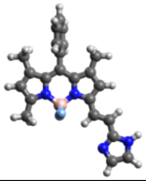	508.06<br>572.51	1.03	0.83
BD3R^3_c	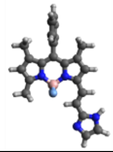	482.65<br>510.03	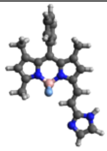	525.24<br>585.64	3.54	2.17
**Substitution R^4**						
BD1R^4_a	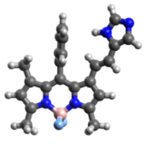	450.28<br>474.28	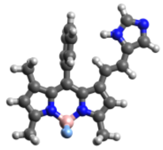	531.44<br>579.99	0.00	0.00
BD1R^4_b	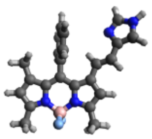	453.19<br>469.36	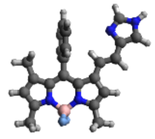	530.99<br>567.62	0.11	1.08
BD1R^4_c	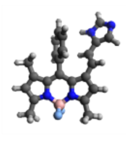	464.30<br>478.54	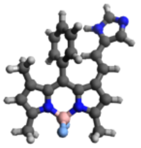	622.65<br>640.83	4.57	2.64
BD2R^4_a	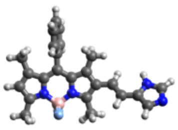	455.55<br>468.64	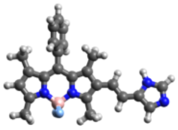	583.81<br>543.01	0.00	0.45
BD2R^{4}m4_b	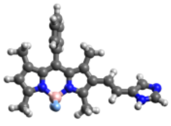	456.69<br>468.57	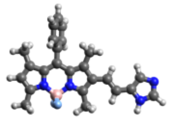	606.28<br>549.34	0.66	0.34

BD2R^4_c	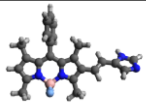	456.64<br>471.07	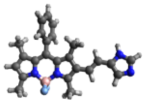	603.13<br>563.02	0.75	0.00
BD3R^4_a	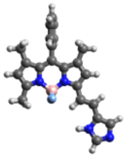	468.83<br>499.43	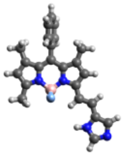	503.32<br>570.04	0.00	0.00
BD3R^4_b	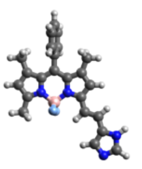	471.97<br>502.52	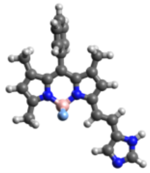	509.54<br>577.16	1.56	1.09
BD3R^4_c	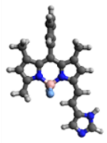	485.73<br>515.42	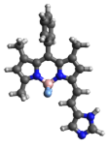	524.35<br>588.31	2.90	1.31

Table S2. Calculated spectroscopic data (λ_{abs} and λ_{em}, nm; $\Delta\bar{\nu}$, cm^{-1}) of neutral and deprotonated forms of pyrrole derivatives in chloroform (M062x/6-31+G(d,p)). Upper lines refer to data obtained by **SS** approach; bottom lines refer to data obtained by **LR** approach.

	**neutral form**	**$\lambda_{abs/}$**	**λ_{em}**	**$\Delta\bar{\nu}$**	**deprotonated form**	**λ_{abs}**	**λ_{em}**	**$\Delta\bar{\nu}$**
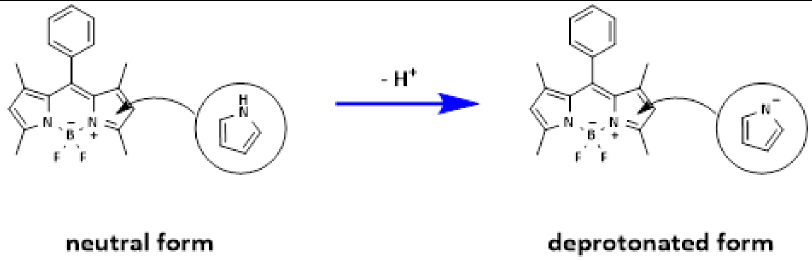 								
1	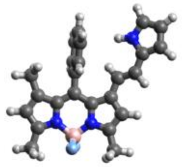	491.49<br>492.77	575.29<br>618.81	2964<br>4133	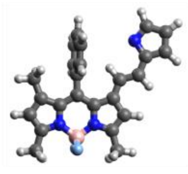	575.73<br>645.65	639.65<br>770.37	1739<br>2511
	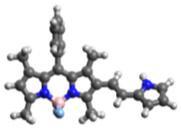	496.27<br>480.03	704.23<br>582.77	5950<br>3673	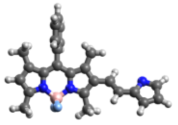	631.07<br>331.09	922.76<br>441.96	5009<br>7577
	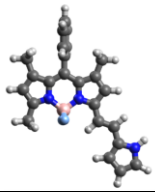	488.62<br>517.83	530.98<br>601.39	1633<br>2683	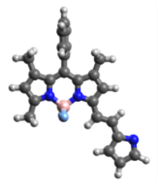	514.98<br>577.42	563.17<br>694.51	1661<br>2919
**2**	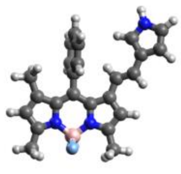	480.03<br>482.92	572.61<br>603.32	3368<br>4132	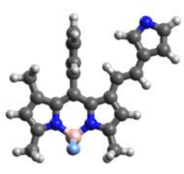	562.58<br>631.57	641.19<br>764.16	2179<br>2748

	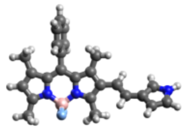	478.91<br>475.22	675.60<br>575.52	6079<br>3667	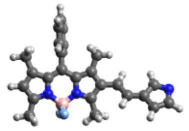	593.24<br>329.59	877.08<br>442.04	5456<br>7719
	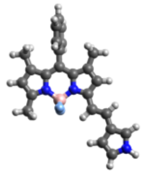	473.07<br>500.62	509.44<br>571.00	1508<br>2462	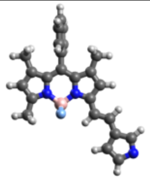	501.43<br>562.31	548.48<br>671.92	1711<br>2901

Table S3. Calculated spectroscopic data (λ_{abs} and λ_{em}, nm; $\Delta\bar{\nu}$, cm^{-1}) of neutral, protonated and deprotonated forms of imidazole derivatives in chloroform (M062x/6-31+G(d,p)). Upper lines refer to data obtained by **SS** approach; bottom lines refer to data obtained by **LR** approach.

	**cation**	λ_{abs}	λ_{em}	$\Delta\bar{\nu}$	**neutral**	λ_{abs}	λ_{em}	$\Delta\bar{\nu}$	**Anion**	λ_{abs}	λ_{em}	$\Delta\bar{\nu}$
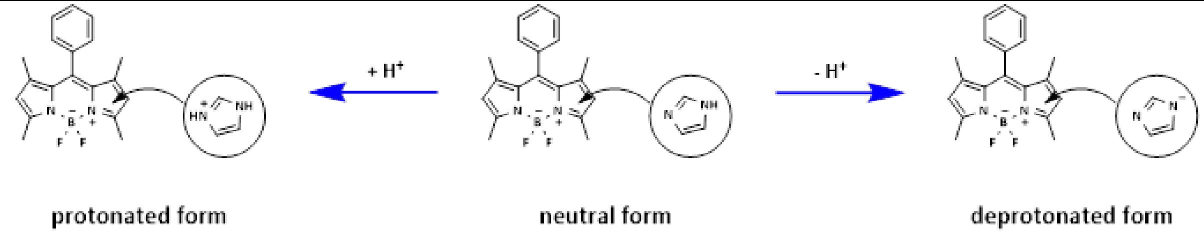												
3	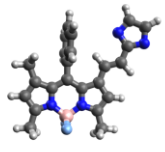	438.8<br>473.9	481.9<br>548.9	2044<br>2885	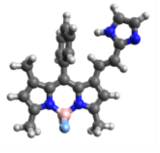	445.4<br>470.4	532.6<br>576.2	3676<br>3902	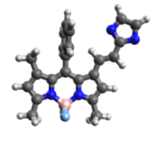	541.6<br>609.8	618.9<br>738.3	2305<br>2856
	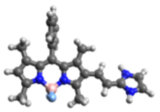	436.5<br>446.8	454.5<br>490.0	912<br>1975	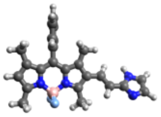	448.3<br>465.3	554.9<br>529.8	4286<br>2619	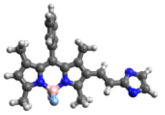	547.8<br>414.1	820.5<br>436.9	6067<br>4873
	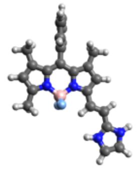	459.5<br>489.4	499.9<br>579.8	1760<br>2885	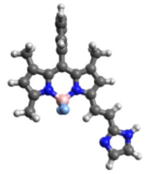	469.3<br>498.0	508.1<br>572.5	1626<br>2613	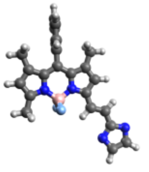	499.9<br>566.7	548.5<br>673.4	1774<br>2796
4	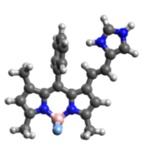	432.9<br>464.9	466.32<br>528.95	1655<br>2602	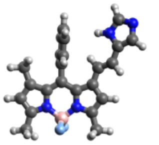	450.3<br>474.2	531.4<br>579.9	3391<br>3843	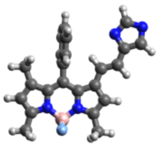	529.6<br>596.6	604.6<br>718.5	2345<br>2843
	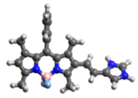	443.0<br>449.9	464.74<br>488.60	1054<br>2602	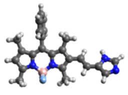	455.6<br>468.6	583.8<br>543.0	4822<br>3843	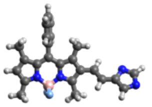	534.9<br>302.6	790.4<br>354.7	6042<br>2843
	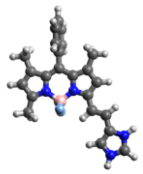	456.9<br>482.2	486.92<br>549.20	1351<br>2532	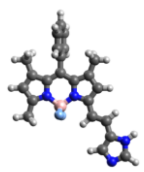	471.9<br>502.5	509.5<br>577.2	1563<br>2574	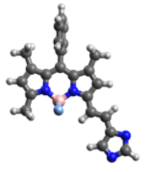	495.5<br>559.5	542.1<br>662.7	1733<br>2783
**1,3,5,7-Tetramethyl-8-phenyl-4,4-difluoroboradiazaindacene**												

	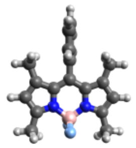	413.<br>436.	429.4<br>471.9	875<br>1732	

Table S4. Frontier molecular orbitals of studied dyes and orbital energy (eV) for ground and excited states.

		Ground state				Excited state			
		Conf.	HOMO	LUMO	$\Delta E_{H\text{-}L}$	Conf.	HOMO	LUMO	$\Delta E_{H\text{-}L}$
ortho	Neutral	1_a 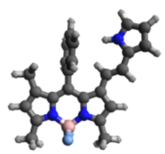	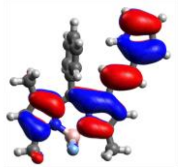	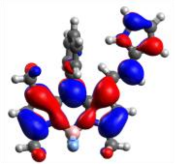	4.222	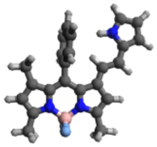	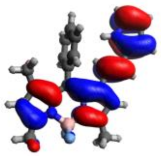	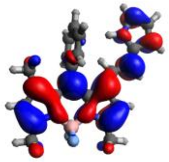	3.687
	Deprotonat ed	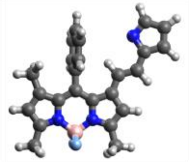	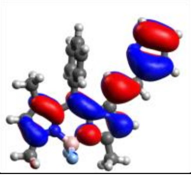	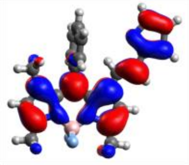	3.466				
	Neutral	2_a 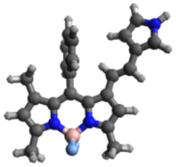	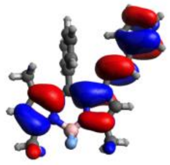	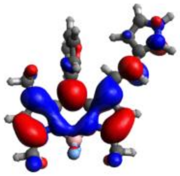	4.345	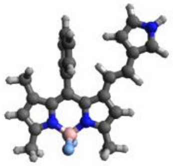	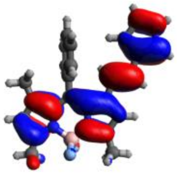	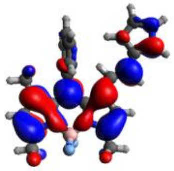	3.831
	Deprotonat ed	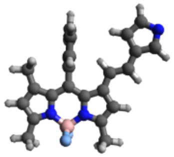	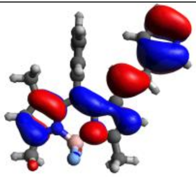	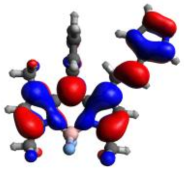	3.581				
	Neutral	3_a 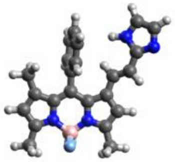	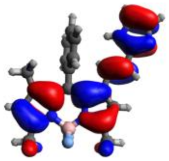	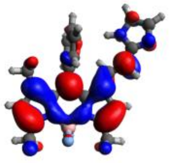	4.435	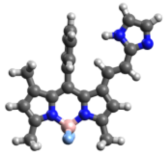	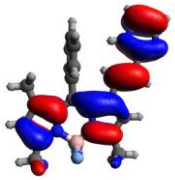	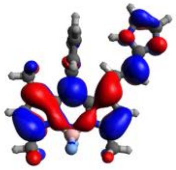	3.874

	Deprotonated	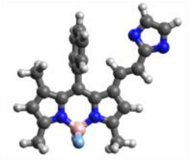	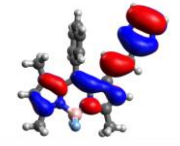	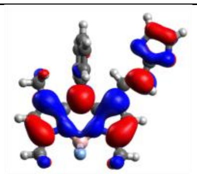	3.578				
	Protonated	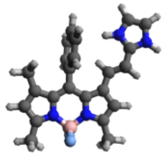	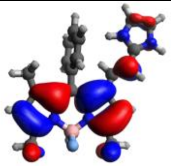	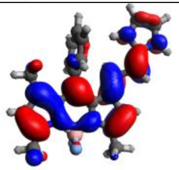	4.451				
	Neutral	4_a 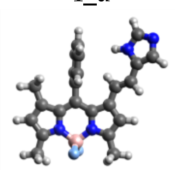	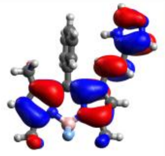	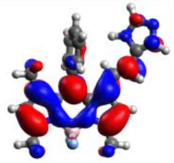	4.395	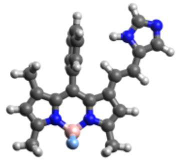	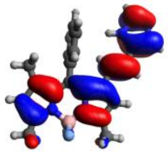	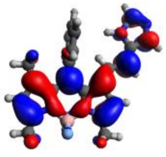	3.858
	Deprotonated	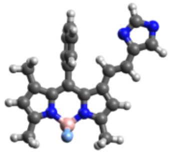	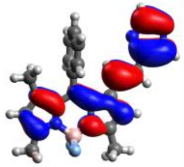	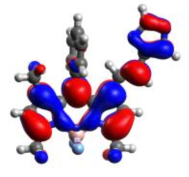	3.661				
	Protonated	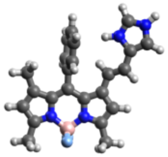	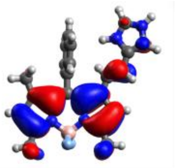	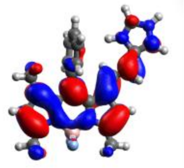	4.512				
Meta	Neutral	1_a 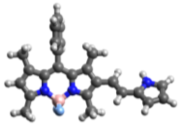	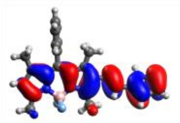	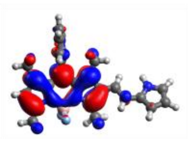	4.279	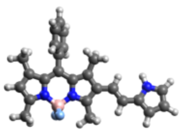 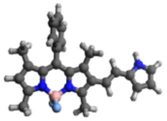	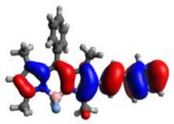 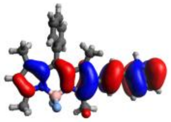	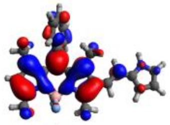 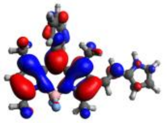	3.731<br>3.601
	Deprotonated	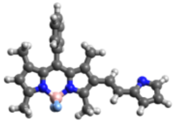	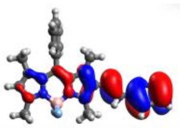	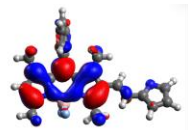	3.198				

	Neutral	2_a 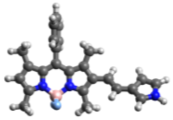	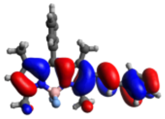	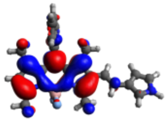	4.371	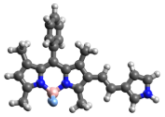 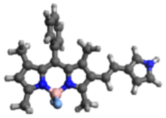	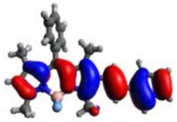 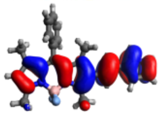	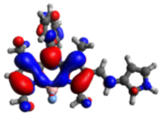 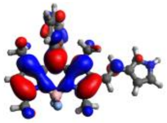	3.835<br>3.801
	Deprotonated	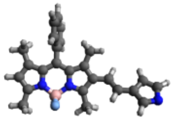	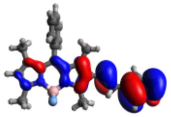	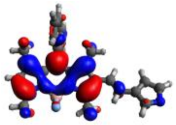	3.359				
	Neutral	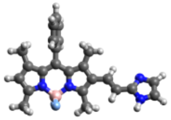 3_a	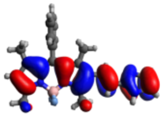	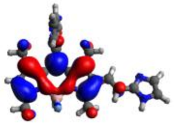	4.444	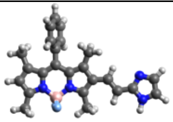 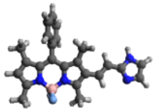	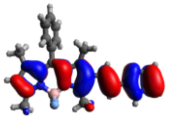 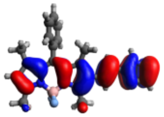	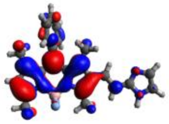 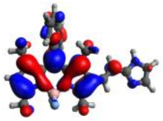	4.033<br>3.907
	Deprotonated	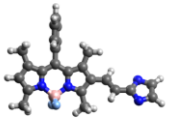	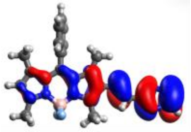	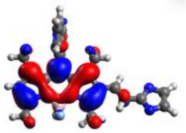	3.499				
	Protonated	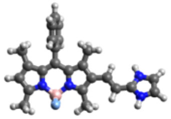	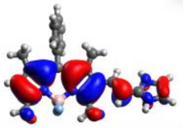	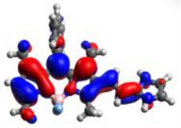	4.692				
	Neutral	4_a 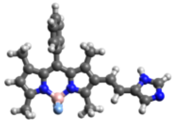	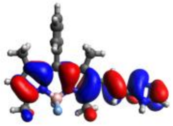	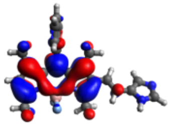	4.439	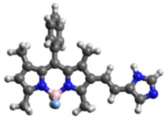 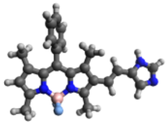	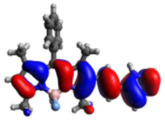 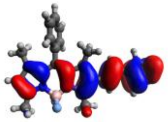	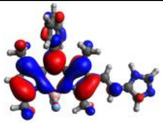 - 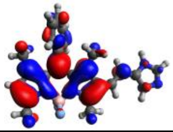	3.928<br>3.834

	Deproton ated	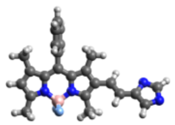	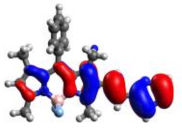	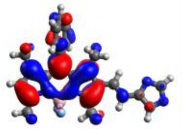	2.972				
	Protonate d	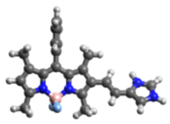	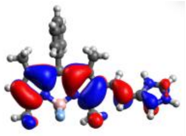	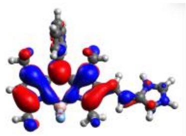	4.667				
Para	Neutral	1_a 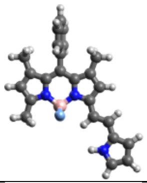	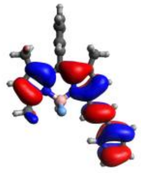	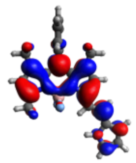	4.086	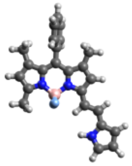	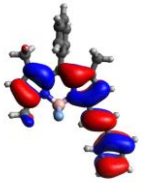	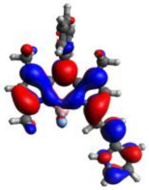	3.781
	Deprotonat ed	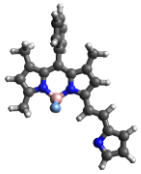	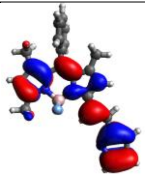	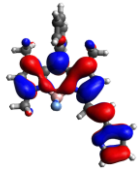	3.666				
	Neutral	2_a 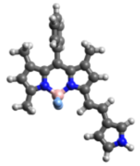	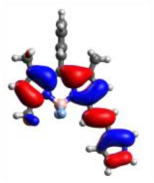	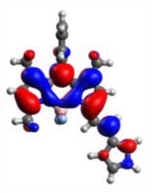	4.193	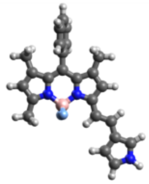	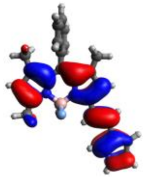	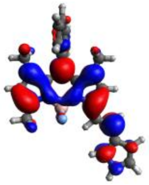	3.895
	Deprotonated	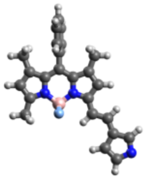	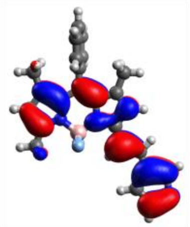	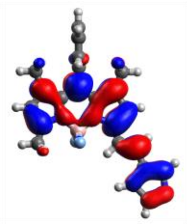	3.768				
	Neutral	3_a 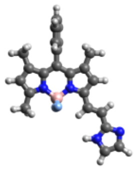	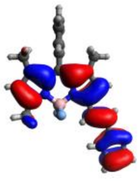	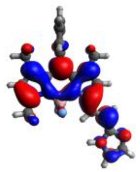	4.226	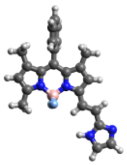	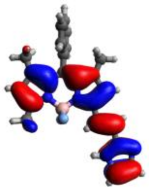	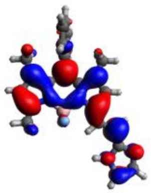	3.905

	Deprotonated	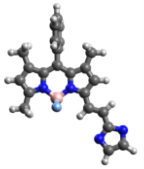	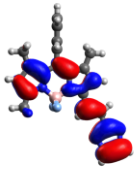	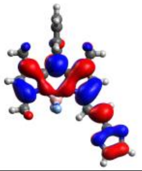	3.721				
	Protonated	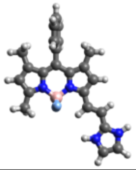	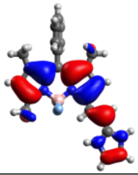	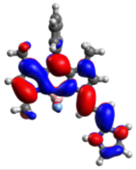	4.323				
	Neutral	4_a 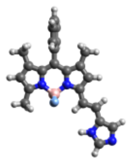	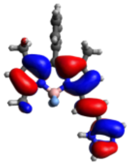	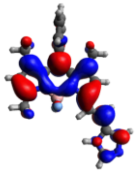	4.204	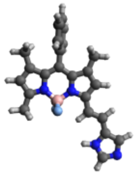	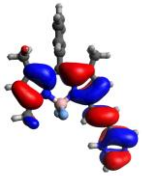	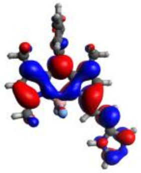	3.892
	Deprotonated	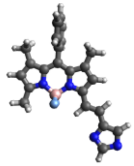	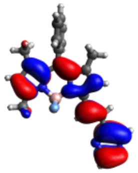	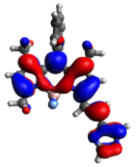	3.796				
	Protonated	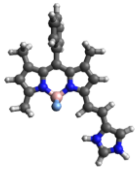	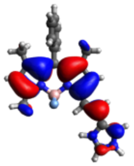	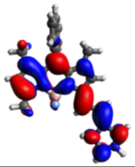	4.310				

Table S5. Calculated spectroscopic data (λ_{abs} and λ_{em}, nm; $\Delta\bar{\nu}$, cm^{-1}) of neutral and deprotonated forms of pyrrole derivatives in chloroform (M062x/6-31+G(d,p)). Upper lines refer to data obtained by **SS** approach; bottom lines refer to data obtained by **LR** approach.

	**R**	λ_{abs}, nm	λ_{em}, nm	$\Delta\bar{\nu}$, cm^{-1}	**R**	λ_{abs}, nm	λ_{em}, nm	$\Delta\bar{\nu}$, cm^{-1}	**R**	λ_{abs}, nm	λ_{em}, nm	$\Delta\bar{\nu}$, cm^{-1}
BD1	1_a	491.49<br>492.77	575.29<br>618.81	2964<br>4133	1_b	504.64<br>497.95	587.34<br>626.57	2791<br>4122	1_c	518.99<br>502.62	672.09<br>683.34	4389<br>5262
	2_a	474.73<br>480.33	559.15<br>591.28	3180<br>3907	2_b	480.03<br>482.92	572.61<br>603.32	3368<br>4132	2_c	505.52<br>476.52	696.07<br>678.31	5415<br>6243
	3_a	445.39	532.58	3781	3_b	451.24	544.07	3781	3_c	460.34	621.13	5624

		470.43	576.21	3902		471.23	576.98	3889		476.08	633.67	5224
	4_a	450.28<br>474.28	531.44<br>579.99	3391<br>3843	4_b	453.19<br>469.36	530.99<br>567.62	3233<br>3688	4_c	464.30<br>478.54	622.65<br>640.83	5477<br>5292
BD2	1_a	496.27<br>480.03	704.23<br>582.77	5950<br>3673	1_b	508.60<br>482.99	730.43<br>591.87	5971<br>3809	1_c	499.58<br>484.5	715.19<br>605.33	6034<br>4120
	2_a	479.68<br>475.77	668.12<br>572.87	980<br>3563	2_b	478.13<br>477.61	661.71<br>584.14	5802<br>3818	2_c	478.91<br>475.22	675.60<br>575.52	6079<br>3667
	3_a	453.90<br>467.39	581.83<br>535.45	2163<br>2720	3_b	455.51<br>471.52	580.69<br>550.28	4732<br>3035	3_c	448.32<br>465.25	554.94<br>529.81	4286<br>2619
	4_a	455.55<br>468.64	583.81<br>543.01	4823<br>2922	4_b	456.70<br>468.57	606.28<br>549.34	2028<br>3138	4_c	456.64<br>471.07	603.13<br>563.02	5319<br>3467
BD3	1_a	484.70<br>514.17	525.07<br>594.17	763<br>2619	1_b	488.62<br>517.83	530.98<br>601.39	1633<br>2683	1_c	504.72<br>532.94	547.06<br>613.84	1533<br>2473
	2_a	473.07<br>500.62	509.44<br>571.00	1509<br>2462	2_b	475.84<br>503.31	513.98<br>575.93	1148<br>2505	2_c	495.33<br>521.36	536.33<br>595.99	1543<br>2402
	3_a	467.69<br>496.90	504.96<br>569.35	1514<br>2561	3_b	469.28<br>498.00	508.06<br>572.51	1627<br>2613	3_c	482.65<br>510.03	525.24<br>585.64	1680<br>2531
	4_a	468.83<br>499.43	503.32<br>570.04	1462<br>2480	4_b	471.97<br>502.52	509.54<br>577.16	1562<br>2573	4_c	485.73<br>515.42	524.35<br>588.31	1516<br>2404

Table S6. Calculated spectroscopic data of conformers referring to global energy minimum of studied dye for different solvents (M062x/6-31+G(d,p)).

			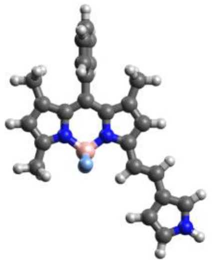 BD3R^2_a				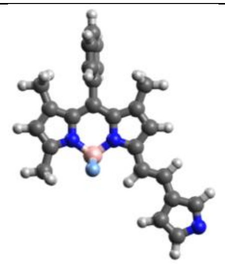 BD3R^2_deprotonated			
			λ_{abs}, nm		λ_{em}, nm		λ_{abs}, nm		λ_{em}, nm	
**N**	**Solvent**	**ε**	**LR**	**SS**	**LR**	**SS**	**LR**	**SS**	**LR**	**SS**
1	Cyclohexane	2.017	498.60	471.80	536.12	504.06	566.84	513.87	640.95	569.98
2	Toluene	2.374	502.23	472.36	543.82	505.16	571.01	511.51	648.36	564.59
3	Diethyl ether	4.240	495.56	472.49	567.34	509.02	555.29	502.25	668.65	550.45
4	Chloroform	4.711	500.62	473.07	571.00	509.44	562.31	501.43	671.92	548.48
5	Ethyl acetate	5.987	497.00	472.98	578.54	510.81	555.04	498.21	678.59	545.28
6	THF	7.426	498.94	473.34	584.42	511.69	556.66	496.19	683.71	542.77
7	Dichloromethane	8.930	500.08	473.59	588.82	512.38	557.36	494.63	687.48	541.00
8	Butanol	17.332	499.15	473.88	600.41	514.29	553.25	490.02	697.42	536.95
9	Propanol	20.524	498.45	473.89	602.49	514.66	551.74	489.12	699.24	536.33
10	Acetone	20.493	497.01	473.75	602.48	514.71	549.63	488.97	699.22	536.39
11	Ethanol	24.852	497.22	473.84	604.51	515.03	549.45	488.19	700.95	535.74
12	Methanol	32.613	495.44	473.74	606.85	515.47	546.32	487.07	702.93	535.09
13	DMF	37.219	501.02	474.31	607.80	515.43	554.19	487.33	703.74	534.62
14	Acetonitrile	35.688	496.35	473.85	607.51	515.55	547.49	486.91	703.50	534.87
15	DMSO	46.826	500.38	474.29	609.19	515.68	552.92	486.71	704.90	534.23
16	Water	78.355	495.86	473.93	611.60	516.35	545.87	485.32	706.79	533.77

Table S6 (continuation). Calculated spectroscopic data of conformers referring to global energy minimum of studied dye in different solvents (M062x/6-31+G(d,p)).

			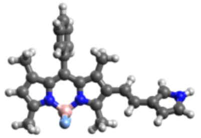 BD2R^2_a				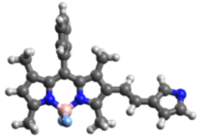 BD2R^2_deprotonated			
			λ_{abs}, nm		λ_{em}, nm		λ_{abs}, nm		λ_{em}, nm	
**Lp.**	**Solvent**	**e**	**LR**	**SS**	**LR**	**SS**	**LR**	**SS**	**LR**	**SS**
1	Cyclohexane	2.017	473.90	471.67	567.38	599.37	410.21	764.82	427.74	970.54
2	Toluene	2.374	476.27	476.42	568.92	611.00	341.10	738.14	449.34	937.70
3	Diethyl ether	4.240	472.00	472.86	574.55	670.54	329.54	472.86	442.28	670.54
4	Chloroform	4.711	475.22	478.91	575.52	675.60	329.59	593.24	442.04	877.08
5	Ethyl acetate	5.987	472.82	475.37	577.56	701.27	326.35	547.44	441.72	870.08
6	THF	7.426	474.03	478.29	579.21	717.89	325.11	535.05	441.55	858.99
7	Dichloromethane	8.930	474.71	480.22	580.46	731.39	324.18	525.27	441.45	851.71
8	Butanol	17.332	474.08	480.18	583.82	775.77	321.11	487.74	441.24	839.37
9	Propanol	20.524	473.64	479.44	584.43	785.20	321.11	479.76	441.24	838.05
10	Acetone	20.493	472.74	477.52	584.42	787.25	320.14	474.69	441.19	839.86
11	Ethanol	24.852	472.87	477.96	585.02	795.46	318.89	477.96	441.12	795.46
12	Methanol	32.613	471.78	475.98	585.70	808.36	318.07	457.37	441.09	837.36
13	DMF	37.219	475.28	483.75	585.98	803.72	319.04	474.47	441.08	829.69
14	Acetonitrile	35.688	472.35	477.16	585.9	809.79	318.17	459.06	441.09	835.64
15	DMSO	46.826	474.89	482.95	586.39	810.78	410.21	468.86	427.74	829.21
16	Water	78.355	472.12	476.97	587.05	828.10				

Table S6 (continuation). Calculated spectroscopic data of conformers referring to global energy minimum of studied dye for different solvents (M062x/6-31+G(d,p)).

			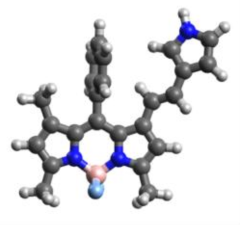 BD1R^2_a				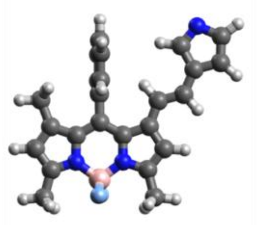 BD1R^2_deprotonated			
			λ_{abs}, nm		λ_{em}, nm		λ_{abs}, nm		λ_{em}, nm	
**N**	**Solvent**	**ε**	**LR**	**SS**	**LR**	**SS**	**LR**	**SS**	**LR**	**SS**
1	Cyclohexane	2.0165	478.94	471.39	573.46	559.02	642.34	583.03	442.44	2706.7
2	Toluene	2.3741	482.53	475.81	579.24	561.21	646.60	579.96	446.60	2524.3
3	Diethyl ether	4.2400	478.79	475.37	599.89	572.01	624.23	563.80	766.11	648.6
4	Chloroform	4.7113	482.92	480.04	603.32	572.61	631.57	562.58	764.16	641.2
5	Ethyl acetate	5.9867	480.52	478.38	610.65	576.18	621.45	555.55	767.41	633.6
6	THF	7.4257	482.33	480.94	544.26	431.22	621.35	551.03	765.71	622.6
7	Dichloromethane	8.9300	483.43	482.63	621.36	579.86	620.51	547.37	766.71	616.8
8	Butanol	17.332	483.25	483.56	632.92	583.34	611.51	535.55	776.33	609.5
9	Propanol	20.524	482.78	483.23	635.3	584.24	609.05	533.08	778.40	608.6
10	Acetone	20.493	481.66	481.97	635.28	584.44	606.90	532.50	778.35	608.6
11	Ethanol	24.852	481.89	482.40	637.54	585.13	605.80	530.38	713.48	555.4
12	Methanol	32.613	480.60	481.23	640.88	587.27	601.64	527.02	712.35	558.8
13	DMF	37.219	484.95	486.27	641.95	586.86	609.08	528.49	810.13	413.8
14	Acetonitrile	35.688	481.32	482.06	641.68	587.44	615.77	534.32	861.43	690.8
15	DMSO	46.826	484.49	485.87	643.57	587.44	607.12	526.67	785.25	605.6
16	Water	78.355	481.06	482.14	646.12	588.84	598.98	522.04	786.71	604.2
